# N-Gene and T-Gene deregulation networks: a data-driven causal framework for the analysis of gene interventions in cancer

**DOI:** 10.3389/fsysb.2026.1861211

**Published:** 2026-07-02

**Authors:** Frank Castel, Roberto Herrero, Jean Pierre Gómez, Gabriel Gil, Augusto Gonzalez

**Affiliations:** 1 Institute of Cybernetics, Mathematics and Physics, Havana, Cuba; 2 Faculty of Mathematics and Computer Science, University of Havana, Havana, Cuba

**Keywords:** clonal evolution, deregulation networks, gene expression data, probabilistic theory of causation, gene interventions in cancer, somatic evolution

## Abstract

**Background:**

Current gene regulatory networks are limited by incomplete functional annotation and the difficulty of inferring causal relationships from expression data. Here we introduce Gene Deregulation Networks (GDNs), a new structure in which a directed link from gene C to gene E indicates that a deregulation of C makes a deregulation of E more probable. GDNs are inferred from expression data using a probabilistic theory of causation, without requiring prior biological knowledge.

**Methods:**

Using previously defined N- and T-genes with exclusive expression intervals for normal tissue and tumors, respectively, we construct separate GDNs for normal and for tumor tissues. Data are TCGA RNA-Seq *bulk* profiles from five cancer types. Links are identified via the Loevinger coefficient and pruned with Reichenbach-type and Mokken tests. We then project each sample onto its corresponding Gene Deregulation Network to visualize the deregulation cascades that have occurred. Finally, we define a simple dynamics: spontaneous evolution follows the direction of the GDN edges; interventions (e.g., gene knockdown) acting against spontaneous evolution induce cascades along the reversed network.

**Results:**

The GDNs are represented by sparse, directed acyclic graphs. Genes with low deregulation frequency have high out-degrees, suggesting they act as upstream regulators. High-frequency genes have high in-degrees, indicating they are convergence points of cascades. Projecting samples onto the T-GDN reveals that early tumors rely mostly on spontaneous T-gene activations, whereas advanced tumors show wide, branching cascades. The N-GDNs show consistent size and structure across distinct tissues revealing similar protective machinery against tumor formation. In contrast, the T-GDNs quantitatively differ from tissue to tissue indicating different levels of transcriptional reprogramming. Simulated knockdown of *EPHA10* and of an 8-gene panel illustrates how the network topology determines whether an intervention can be resisted by the tumor. A reported experiment on POM121 knockdown in two prostate cancer cell lines qualitatively confirms the predicted directionality of cascades.

**Conclusion:**

GDNs provide a robust, scalable, and annotation-free framework to understand cancer onset and progression. The separation into N- and T-GDNs, connected by NT-genes, offers a systematic basis for studying carcinogenesis and designing targeted therapies.

## 1 Introduction

### 1.1 The challenge of large-scale causal inference in cancer

Understanding how gene expression deregulations propagate in cancer is a central goal of systems biology. Experimental evidence shows that perturbing a single gene can trigger extensive transcriptional changes (Miranda et al., 2023), implying that realistic networks must include thousands of genes and their interconnections. However, current gene regulatory networks (GRNs) are typically limited to dozens of genes because they rely on detailed functional annotations or on co-expression-based methods that cannot distinguish correlation from causation (Karlebach and Shamir, 2008; Emmert-Streib et al., 2014).

### 1.2 What is a gene deregulation network (GDN)?

We introduce a different concept: Gene Deregulation Networks (GDNs). Instead of asking “do genes C and E interact functionally?”, we ask a simpler, purely probabilistic question: *“Does the probability that gene E is deregulated increase when gene C is deregulated?”* If yes, we add a directed link C → E. This notion is directly grounded in probabilistic theory of causation (Suppes, 1970; Reichenbach, 1971; Cartwright, 1979): a cause raises the probability of its effect.

Crucially, GDNs do not require any prior biological knowledge - no pathways, no Gene Ontology, no known interactions. They are inferred directly from expression data, making them applicable to any cancer type, even for poorly annotated genes.

### 1.3 Advantages over existing GRN inference methods

Many causal discovery algorithms exist (e.g., PC, FCI, LiNGAM) that infer directed graphs from observational data using conditional independence or non-Gaussianity (Spirtes et al., 2000; Shimizu et al., 2006; Triantafillou et al., 2017; Zhang and Hyvärinen, 2009). Other popular GRN inference methods, such as GENIE3, are not causal but rather predict regulatory importance based on machine learning (Huynh-Thu et al., 2010). Both families face two major limitations when applied to cancer genomics:

- Scalability: Most causal algorithms become computationally infeasible for ∼20,000 genes and hundreds of samples.
- Causal interpretability: GENIE3 and similar scalable methods do not provide causal interpretation.
- Dependence on prior biological information: Obtaining robust large-scale networks with GENIE3-like methods often requires incorporation of prior biological information.
- Validation: The dream challenges (Marbach et al., 2012) provided gold standards for *E. coli* (∼4,000 genes) and for small synthetic networks, but no gold standard exists for human cancer.

Our GDN framework is designed to be scalable (we handle 60,000 transcripts) and directly usable for two specific tasks that no other GRN method can tackle:

1. Cascade visualization: By projecting a sample’s deregulation profile onto the GDN, one can see exactly which genes were spontaneously activated and how the deregulation propagated through the network. This provides a sample-specific map of the “evolutionary history” of that tumor.
2. Trivial dynamics: Spontaneous evolution (e.g., tumor onset and progression) follows the direction of the GDN edges. Interventions (e.g., gene knockdown) acting against spontaneous evolution induce cascades along the reversed network. No complex differential equations or kinetic parameters are needed - the network itself encodes the directionality of influence.

### 1.4 Why separate N- and T-networks?

We recently introduced N- and T-genes, which are characterized by exhibiting expression intervals exclusive to normal tissues and tumors, respectively (Gil et al., 2025; Gil Perez et al., 2024). Somatic evolution of normal tissue involves only the progressive deactivation of N-genes, while clonal evolution of tumors involves only the progressive activation of T-genes (Gil Perez et al., 2024). Therefore, we construct separate GDNs: the N-GDN from normal samples, and the T-GDN from tumor samples. This separation avoids mixing two different biological processes.

Besides, there is a significant reduction of dimensionality. In prostate adenocarcinoma, for example, the N-GDN involves around 1,000 genes, whereas in the T-GDN there are around 6,000 genes involved. The remaining 53,000 transcripts in the data are excluded from the dynamics.

NT-genes (present in both networks) act as bridges during the normal-to-tumor transition or interventions inducing partial phenotype reversal (Gil et al., 2026).

### 1.5 A note on bulk RNA-Seq data and the need for validation

We use TCGA bulk RNA-Seq data because it provides the largest available cohort sizes. On the other hand, the use of single-cell data is a more complex process, requiring the integration of effects across different cell types. It is well known that bulk data mix malignant, stromal and immune cells. Our causal inference scheme, when applied to bulk data, should be regarded as an effective cross-sectional analysis. We fully acknowledge these limitations.

We do not claim that our GDNs represent true biological causality in a strict interventionist sense. Rather, they generate testable hypotheses about robust, directional correlations among gene deregulations. The combinatorial space of possible causal relations among genes in genome-wide expression data is far too large to be explored exhaustively through targeted experiments alone. In this sense, we take our *in silico* framework as a pragmatic large-scale pre-screening strategy for prioritising candidate causal relations for experimental validation.

Validation must ultimately come from perturbation experiments (e.g., knockdowns) and, in the future, from single-cell time-series data. In this work, we provide a preliminary comparison with a *POM121* knockdown experiment (Section 3.7) to motivate further validation of our GDN results. However, comprehensive validation of these causal hypotheses constitutes a substantial research program in its own right, spanning multiple layers of analysis and methodological approaches, including computational studies and targeted experimental interventions, and therefore remains an ongoing line of research extending beyond the present study.

## 2 Methods

### 2.1 Data acquisition

TCGA level 3 RNA-Seq expression data (Tomczak et al., 2015) (Data version: GDC Data Release 19.0 (September 2019), Access date: October 2019) were obtained for five cancer localizations: prostate adenocarcinoma (PRAD) (Cancer Genome Atl as Research Network, 2015), head and neck squamous carcinoma (HNSC) (Cancer Genome Atlas Network, 2015), lung adenocarcinoma (LUAD) (Cancer Genome Atlas Research Network, 2014), uterine corpus endometrial carcinoma (UCEC) (Cancer Genome Atlas Research Network et al., 2013), and lung squamous cell carcinoma (LUSC) (Cancer G enome Atlas Research Network, 2012). For each localization, we downloaded both normal and primary tumor samples. The sample counts were: PRAD (52 normal, 499 tumor), HNSC (44 normal, 502 tumor), LUAD (59 normal, 535 tumor), UCEC (23 normal, 552 tumor), LUSC (49 normal, 502 tumor). These numbers reflect the availability at the time of download.

Normal samples are derived from adjacent pathological normal tissue in some cancer patients. These patients contribute both normal and tumor samples to the dataset. In that case, we refer to the normal and tumor samples as paired samples.

Expression values were provided in FPKM units (Fragments Per Kilobase of transcript per Million mapped reads).

### 2.2 Definition of N-, T- and NT-genes and expression discretization

The concept of N- and T-genes relies on identifying expression intervals that are exclusive to normal samples or to tumor samples, respectively. For each gene, we construct paired histograms for normal and tumor samples (examples are shown in Section S0 of the Supplementary Material). We then discretize the continuous expression values into three states, following the procedure introduced in (Gil et al., 2025; Gil Perez et al., 2024):

- e = −1 (N-active) if and only if expression falls in an interval that contains only normal samples (no tumor samples). Such an interval must contain at least a minimum number of normal samples to be statistically significant.
- e = 1 (T-active) if and only if expression falls in an interval that contains only tumor samples. Again, a minimum number of tumor samples is required.
- e = 0 (inactive) otherwise, i.e., if expression falls in the common interval where both normal and tumor samples coexist, or in intervals that are not statistically exclusive.

Note that the same discrete value e = 1 may correspond to over-expression for a tumor-above gene, or under-expression for a tumor below gene, or expression in the outside interval for a tumor-outside gene (Gil et al., 2025; Gil Perez et al., 2024). Conversely, for N-genes, e = −1 corresponds to under-expression in normal-below genes, over-expression in normal-above genes, and expression outside an interval in normal-outside genes.

To determine statistical significance, we apply a one-sided Fisher exact test. In practice, we require that candidate exclusive intervals contain at least 5% of total normal samples for N-genes and at least 10% of total tumor samples for T-genes. Computational experiments indicate that these minimum-frequency requirements already imply statistical significance under the conventional p-value threshold of 0.01, and therefore do not constitute arbitrary empirical cutoffs. When converting proportions into sample counts, conservative upward rounding is applied. For example, for PRAD, this means that an N-only interval must contain at least three normal samples, whereas a T-only interval must contain at least 50 tumor samples. The asymmetry (5% vs. 10%) reflects the larger number of tumor samples in TCGA while maintaining approximately the same statistical significance level for both N- and T-genes across datasets from all cancer types.

Genes that pass the significance test for N-only intervals are classified as N-genes; those that pass for T-only intervals are T-genes. Genes satisfying both criteria are NT-genes. All remaining genes, i.e., genes with neither significant N-only nor T-only intervals, are classified as O-genes. In PRAD, this procedure yielded 1097 N-genes, 6138 T-genes, and 316 NT-genes, leaving ∼52,000 O-genes. For other cancer types, similar numbers are reported in Table 1.

**TABLE 1:** Global and local properties of T-GDNs and N-GDNs for five cancer types.

​	PRAD	HNSC	LUAD	UCEC	LUSC
T-GDN					
Number of T-genes	6,138	9,340	17,037	16,469	18,596
Rank (number of nodes)	6,138	9,340	17,037	16,468	18,594
Sparsity	3 × 10^−3^	2 × 10^−3^	2 × 10^−3^	2 × 10^−3^	3 × 10^−3^
Max freq_T	0.74	0.89	0.98	1	1
Mean diameter	2.6	2.4	2.5	2.4	2.3
Isolated nodes (in-degree = 0 and out-degree = 0)	129	114	19	7	1
Mean in-deg = mean out-deg	18.1	21.3	34.5	33.3	56
Max in-deg	239	385	916	1,532	1,028
Max out-deg	281	161	264	280	403
Child-less nodes (out-deg = 0)	513	586	358	245	32
Orphan nodes (in-deg = 0)	1,605	2,919	4,591	4,820	5,216
N-GDN					
Number of N-genes	1,097	1,372	492	1,192	1,269
Rank (number of nodes)	492	766	470	772	1,228
Sparsity	2 × 10^−2^	1 × 10^−2^	1 × 10^−2^	1 × 10^−2^	1 × 10^−2^
Max number of genes in a block node	216	93	4	131	11
Max freq_N	0.31	0.5	0.81	1	0.9
Mean diameter	2.4	2.8	2.6	3.2	2.9
Isolated nodes (in-degree = 0 and out-degree = 0)	1	0	0	1	0
Mean in-deg = mean out-deg	9.8	10.7	7.1	8.9	11.4
Max in-deg	54	53	26	30	43
Max out-deg	163	151	39	63	60
Child-less nodes (out-deg = 0)	93	120	81	86	133
Orphan nodes (in-deg = 0)	9	11	6	4	9

Sparsity = edges/(nodes × (nodes-1)). Max freq, maximum deregulation frequency among nodes. For N-GDN, “Max genes in a block” indicates the size of the largest compressed node.

O-genes never adopt a state that uniquely distinguishes normal from tumor samples. Their activation state is always e = 0. For this later reason O-genes are not further considered in the dynamics, which is described as a series of activation or deactivation events (Gil Perez et al., 2024).

Important note on deregulation: When e = 1 (T-active), a T-gene is regarded as deregulated. However, when an N-gene assumes the value e = 0 (inactive), it is likewise regarded as deregulated. This asymmetry is crucial for interpreting the direction of causality in the N-GDN (where edges represent the propagation of deactivation events) and the T-GDN (where edges represent the propagation of activation events).

The definition of deregulation for N-genes reflects a working hypothesis: all N-genes are activated in the normal state before somatic evolution begins. In practice, all traces of the originally active nodes in a given sample are progressively lost in the course of somatic evolution (Gil Perez et al., 2024).

Examples of N-, T-, NT- and O-genes in PRAD are given in Section S0 of the Supplementary Material.

### 2.3 Construction of gene deregulation networks: causal sufficiency and pruning

We refer the reader to Section S1 of the Supplementary Material for a schematic pipeline of GDN construction and the dynamics following from the GDNs.

Once the sets of N- and T-genes are defined, we construct two separate binary matrices:

- N-GDN data matrix: rows = N-genes, columns = normal samples. Entry = 1 if the N-gene is deregulated (i.e., e = 0, inactive), otherwise 0.
- T-GDN data matrix: rows = T-genes, columns k = tumor samples. Entry = 1 if the T-gene is deregulated (i.e., e = 1, active), otherwise 0.

These matrices are the input to our causal discovery algorithm, implemented in the C++ code CChains (Gomez, 2023; Castel, 2025; Gómez et al., 2026). The algorithm follows the probabilistic theory of causation (Suppes, 1970; Reichenbach, 1971; Cartwright, 1979) and proceeds in four stages.

Stage 1: Prima facie causal sufficiency. For each ordered pair of genes (i, j), we compute:

- v_i_ = frequency of deregulation of i (i.e., proportion of samples where entry = 1 in the data matrix).
- v_j_ = frequency of deregulation of j.
- v_j∣i_ = conditional frequency of deregulation of j given deregulation of i.
- The Loevinger coefficient (Loevinger, 1948):

$$H_{\text{ij}}=\left(v_{\left(j|i\right)}-v_{j}\right)/\left(1-v_{j}\right)$$

A *prima facie* causal sufficiency relation i → j is declared if and only if:

1. 0 < v_i_ ≤ v_j_ <1 (i.e., i is not always absent, j is not always present, and the cause is not rarer than the effect - conditions consistent with probabilistic causal sufficiency and introduced to avoid trivial or reverse directions).
2. H_ij_ > H_0_, where we set H_0_ = 0.5. This means that when i is deregulated, j is deregulated in more than half of the cases beyond its baseline frequency.

The choice H_0_ = 0.5 is not arbitrary. For PRAD data, with minimum deregulation frequencies around 0.1 and ∼500 tumor samples, the Fisher test p-value for a pair of T-genes satisfying H_ij_ = 0.5 is on the order of 10^–18^, indicating that such pairs are extremely unlikely to arise by chance. In contrast, using H_ij_ = 1/8 yields a p-value of 0.15, which would not be significant. Moreover, the statistical significance associated with the Loevinger threshold of H_0_ = 0.5 remains consistently high across a broad range of deregulation frequencies. The stability with respect to frequencies follows from the fact that the frequency of the putative effect participates both in the numerator and denominator of H_ij_. For instance, even when both i and j have deregulation frequencies of 0.95, the condition H_ij_ = 0.5 corresponds to a contingency table with a p-value on the order of 10^–14^.

Stage 2: Pruning spurious links (Reichenbach test (Reichenbach, 1971)). A link i → j that survives stage 1 may still be spurious if it arises from a common cause k (i.e., k → i and k → j). To detect such cases, we condition on the candidate common-cause deregulation status. Specifically, for any third gene k such that k → i and k → j, we compute the Loevinger coefficient by restricting the dataset to the deregulated and non-deregulated states of gene k (denoted by H_ij∣k_ and H_ij∣¬k_, respectively). If H_ij∣k_ and H_ij∣¬k_ both drop below H_0_ for at least one k, the causal relation i → j is considered spurious and the associated link is removed from the GDN. The full mathematical formulation is given in Section S2 Supplementary Material.

Stage 3: Pruning transitive edges (Mokken test of double monotony)**.** Even after removing common-cause artifacts, some links may be redundant due to transitivity: if i → k and k → j are already in the network, the direct link i → j may not represent an independent causal relation. We apply the Mokken test of double monotony (Mokken, 1971), which in our context checks whether the probabilistic inference from a deregulation at i to a deregulation at j can be carried through a deregulation at k, given the chain i → k → j and the link i→j. If so, the direct edge i → j is removed. This test is described in detail in section S2.

Stage 4: Orientation of edges when the frequencies are equal**.** For pairs where vi = vj and the Loevinger coefficients are symmetric (H_ij_ = H_ji_), the direction of an edge passing the first stage is not determined. We use two strategies to orient such edges:

*Mokken orientation of double edges:* For triangles formed by the chain i→k→j and the link i→j containing one or more links in both directions, the verification of the Mokken test allows us to resolve the overall directional structure of the triple and therefore eliminate directionally inconsistent links. For instance, if i→k→j, i→j, and k→i are links in the network, and the ordered triple i,j,k satisfies the Mokken test, we remove the link k→i, effectively orienting the inferred causal relation between i and k.

*Robustness test:* For the remaining double edges (rare cases, six remaining double edges in T-GDN in PRAD, for example), we apply a heuristic test based on the reliability of gene deregulation patterns across samples, originally suggested by Mokken (1971) and adapted to gene expression data in (Gomez, 2023; Castel, 2025). The heuristic is described in section S2.

After these four stages, the remaining directed edges form a sparse directed acyclic graph (DAG) for both the N-GDN and the T-GDN. Empirically, we never observed cycles in the final networks, consistent with the causal sufficiency framework. The sparsity of the network links diminishes by at least one order of magnitude from the edge-addition stage 1 to the completion of the edge-removal stages 2–4.

### 2.4 Handling gene blocks: compression of identical profiles

During the construction of the binary matrices, we noticed that many N-genes exhibit identical deregulation patterns across all normal samples. That is, for a set of genes, the vector of 1/0 entries (across samples) is exactly the same. We call such a set a gene block. In the N-GDN, blocks are very frequent: the largest block in PRAD contains 216 genes, and there are many blocks of smaller size (see Table 1 and (Gil Perez et al., 2024)).

These genes are causally indistinguishable within our framework: any causal relationship that holds for one member of the block holds identically for all others. Therefore, we merge each block into a single representative node in the network. This compression reduces the N-GDN in PRAD from 1,097 genes to only 492 nodes, without any loss of causal information. It also greatly simplifies network visualization and analysis.

Why do blocks occur? We interpret them as evidence for coordinated, multi-gene regulatory modules that are activated or deactivated as a single unit during somatic evolution. This is consistent with the multi-step model of cancer (Frank, 2007), where each “step” may involve simultaneous changes in many genes rather than a single gene.

Note that an alternative explanation could be limited sample size. However, normal-bank subsampling in PRAD (Section S3 of Supplementary Material) shows that the size of the largest block remains in average above 216 genes in a cohort with half of the number of normal samples, suggesting that the gene blocks are not mere sampling artifacts.

### 2.5 Software and post-processing

All steps of GDN construction (Loevinger coefficient calculation, Reichenbach and Mokken tests, orientation heuristics) are implemented in an in-house C++ software named CChains (Gomez, 2023; Castel, 2025; Gómez et al., 2026). CChains takes as input a deregulation binary matrix (genes × samples) and outputs adjacency lists of the resulting directed graph, as well as summary statistics. The code is designed for memory and computational efficiency; it can handle up to 60,000 nodes and 1,000 samples on a standard PC.

For network post-processing and topological analysis, we leverage the NetworkX Python library (Hagberg et al., 2008; Castel et al., 2026). Using NetworkX, we compute:

- In-degree and out-degree distributions.
- Number of isolated nodes (in-degree = out-degree = 0).
- Number of orphan nodes (in-degree = 0) and child-less nodes (out-degree = 0).
- Network diameter (longest shortest path).
- Other metrics.

The complete source code of CChains, along with example input files and a README, is available at https://github.com/gabriel-gil/CChains. A companion repository for the GDN framework, available at https://github.com/fcastelm/GDN, provides analysis code together with CChains example inputs and outputs required to construct, inspect, and reproduce the GDNs presented in this work. The binary deregulation matrix used as input by CChains is generated using a version of our GenePan code, available at https://github.com/gabriel-gil/GenePan (see also (Gil et al., 2025)).

### 2.6 Dynamics: spontaneous evolution vs interventions

One of the main advantages of GDNs is that they enable a simple, topology-driven simulation of both spontaneous evolution and the effects of gene interventions. In some simulations, kinetic parameters (rates) may not be required - only the direction of edges and the initial deregulation state of the sample.

#### 2.6.1 Spontaneous dynamics

In the absence of external perturbations, the system evolves according to the natural direction of the GDN:

- For the T-GDN (tumor samples), spontaneous evolution involves the activation of T-genes. If gene i is active (e = 1), gene j is inactive (e = 0) and there is an edge i → j, then over time j will also become active. This models the propagation of deregulation cascades during tumor progression.
- For the N-GDN (normal samples), spontaneous evolution involves the deactivation of N-genes. If a gene i is deactivated (e = 0), gene j is active (e = −1) and there is an edge i → j in the N-GDN, then j will become deregulated (inactive) over time. This corresponds to loss of normal function.

Note that the deregulation cascade advances in the direction of increasing frequencies in the T-GDN, but in the direction of decreasing frequencies (increasing deactivation frequencies) in the N-GDN.

##### 2.6.1.1 Interventions

Interventions aligned with the spontaneous dynamics (forced activation of a T-gene in a tumor, for example) differ from the spontaneous dynamics only in the rate at which new deregulations occur. We suppose that their rate is greater than the spontanoeus-evolution rate, but this is not a fundamental assumption of our methodology.

However, when the intervention acts against the natural dynamics, such as a forced deactivation of a T-gene, we assume that the perturbation propagates against the direction of the GDN edges.

The argument supporting this assumption is that Loevinger coefficients capture the same statistical dependencies for the direct and reverse processes. For example, in the T-GDN the Loevinger coefficient encoding the *direct* process (namely, H_ij_), whereby the activation of i induces the activation of j, is equal in value to the Loevinger coefficient encoding the *reverse* process (denoted H_¬j¬i_), whereby the deactivation of j induces the deactivation of i. In the last example, H_¬j¬i_ is computed in terms of the conditional and unconditional deactivation frequencies of i and j across tumor samples.

In practice, for a forced deactivation of a T-gene X, we:

1. Set X to the inactive state (e = 0) permanently.
2. Perform a reverse breadth-first search starting from X, following edges against their direction (i.e., from child to parent).
3. All genes reachable by this reverse walk are candidates for forced deactivation. In our simulations, we randomly chose one inactive-child/active-parent pair and deactivate the parent with probability 0.5 (incomplete penetrance).
4. Recurrently continue this process until no more active nodes reached by the reverse cascade are available.

Note that the reverse cascade advances in the direction of decreasing frequencies in the T-GDN, but in the direction of increasing frequencies (decreasing deactivation frequencies) in the N-GDN.

##### 2.6.1.2 Simulation algorithm (Glauber-like discrete time)

We adopt a sequential update scheme analogous to Glauber dynamics (Glauber, 1963). In the T-GDN:

- At each time step t, we have a set of active T-genes.
- Spontaneous step: Randomly select an active node, i. Then select an edge i → j in the GDN, if j is inactive, then j becomes active with probability p_direct_. In intervention simulations, we use also a rate (e.g., p_direct_ = 0.1 per step) to model the tumor’s attempt to recover.
- Forced step (if intervention): In an intervention, we enforce that the target gene(s) or any gene already forced inactive remain inactive. Then, select a node from this set, j, and a reverse edge (j → i). We deactivate the upstream gene with probability p_force_ (usually 0.5 in our simulations).
- The process is iterated for a number of steps (e.g., 100, 1,000). The number of steps is a pseudo-time that correlates with the real progression time, but no absolute calibration is attempted.

A similar procedure applies for the dynamics in the N-GDN.

### 2.7 Validation against experimental knockdown data

To assess whether our T-GDN directionality predictions are consistent with real perturbation experiments, we used data from a recent study (Kirthika et al., 2025) where the transcriptional regulator *POM121* was knocked down in two prostate cancer cell lines (X22RV1 and PC3). The experiment included 4 control replicates and 4 knockdown replicates - a small sample size that is insufficient to define N/T intervals from scratch. Therefore, we relied on the T-gene list derived from TCGA-PRAD data (Section 2.2) and used the following qualitative criterion to detect deactivation in the experiment:

- For each T-gene measured in the experiment, compute the mean log2-fold change (knockdown vs. control). Let Δ_exp_ be this value.
- From TCGA data, compute the mean log2-fold change between normal and tumor samples, Δ_TCGA_ (for the same gene). This represents the typical deregulation magnitude *in vivo*.
- A tumor-above gene is considered activated if - Δ_exp_ < Δ_TCGA_ < - 0.1. Conversely, if Δ_exp_ < Δ_TCGA_ < - 0.1, we consider the gene deactivated in the experiment.
- On the other hand, A tumor-below gene is considered activated if - Δ_exp_ > Δ_TCGA_ > 0.1. Conversely, if Δ_exp_ > Δ_TCGA_ > 0.1, we consider the gene deactivated in the experiment.

This criterion is conservative and hypothesis-generating, not a formal validation. Nevertheless, the results (Section 3.7) show a significant enrichment of deactivations among T-genes with frequency lower than that of *POM121*, which is consistent with the directionality predicted by our GDN (knockdown of *POM121* should preferentially deactivate upstream genes in T-GDN). We also examine N-genes as a negative control, expecting no systematic changes.

### 2.8 Stability and permutation tests

To evaluate the robustness of our GDNs, we performed two complementary tests. Full details are in section S3 Supplementary Material.

#### 2.8.1 Subsampling (stability) test

We evaluated the stability of N-gene and T-gene sets in PRAD, as well as T-GDN edges, under variations in cohort composition and size.

Effect of cohort composition: We created 100 replicates of 52 normal +52 tumor samples (randomly drawn from 499 tumors). For each replicate, we applied the gene discovery algorithm. One replicate served as reference to compute recall and precision. For N-genes recall is high (mode ∼70%) but precision is low (mode <40%). This indicates a robust core of N-genes consistently recovered, but subsampling tumors creates spurious N-genes in some replicates, inflating the replicate-specific sets. For T-genes both recall and precision are high (modes ∼70% and ∼80%, respectively). Tumor-exclusive intervals persist under subsampling due to broad expression distributions, and spurious T-genes cannot arise because normal intervals cannot be extended.

Effect of cohort size: Ten independent replicates (starting at 52 tumors) were progressively enlarged to 468 tumors in steps of 52, keeping all normal samples. T-gene recovery shows recall relative to the reference cohort of 52 tumors that saturates near 75%. Recall relative to the original cohort (499 tumors) approaches 100% as tumor bank size increases. With only half the tumor bank, ∼95% of original T-genes are recovered. Edges at the level of Stage 1 in Section 2.3 (no pruning) were computed. Edge recovery shows a stable core of about 20% of reference-cohort edges recovered regardless of sample size. With half the tumor bank, nearly 75% of original-cohort edges are recovered.

Conclusion: The inferred GDNs are sufficiently stable for identifying candidate gene targets based on topological properties. Although some variation exists, a large portion of the network structure is consistently recovered.

##### 2.8.1.1 Permutation test (null model)

To rule out the possibility that the observed network structure arises purely from marginal frequencies (i.e., from genes that happen to be deregulated at random and independently), we generated 10 permuted T-datasets in PRAD: we randomly shuffled the deregulation vector (the 1/0 pattern) of all genes across samples, preserving the gene’s overall deregulation frequency but breaking any genuine co-occurrence structure. We then ran the full T-GDN inference pipeline on each permuted dataset. In all of the runs, the number of edges retained after pruning was less than 0.02% of the number found in the real data (mean number 22 vs more than 100,000 in real data). This confirms that the GDNs are not artifacts of the frequency thresholds or the causal inference algorithm, but reflect genuine structure in the data.

## 3 Results

### 3.1 The T-GDN in PRAD: global properties

The PRAD T-GDN contains 6,138 genes (nodes) and 110,953 directed edges, with a sparsity of only 0.29% (i.e., 0.29% of all possible directed pairs are present). This indicates that despite the large number of genes, deregulation follows structured, non-random pathways.

**FIGURE 1:**
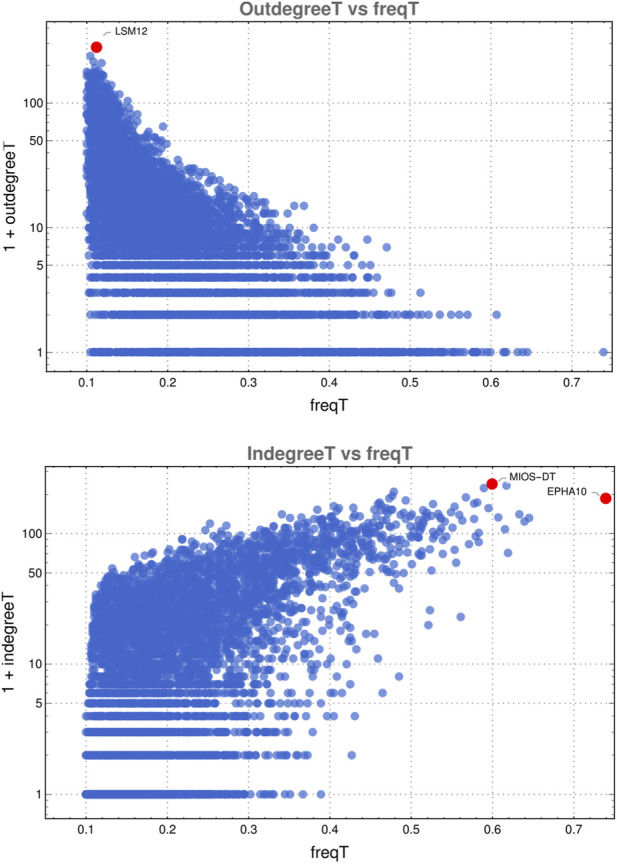
Characterization of the T-GDN in PRAD. Each dot is a gene. Top: In-degree vs. frequency; Bottom: out-degree vs. frequency. Low-frequency genes have high out-degrees; high-frequency genes have high in-degrees. *EPHA10* shows the highest activation frequency (0.74) and a high in-degree (185). *MIOS-DT* exhibits the highest in-degree, 239. A gene in the low-frequency region, *LSM12*, shows the highest out-degree (281).

Key topological patterns (Figure 1; Table 1):

- Genes with low deregulation frequency (< 0.2) tend to have high out-degrees (many downstream effects). Example: the *LSM12* protein coding gene (frequency 0.11, out-degree 281), which has not been clinically tied to prostate cancer. These are candidates for “master regulators” that, when activated, can influence many downstream genes.
- Genes with high deregulation frequency (> 0.4) tend to have high in-degrees (many upstream regulators converge on them). Example: *EPHA10* (frequency 0.74, in-degree 185). These are candidate hubs and potential therapeutic targets, but note that their high in-degree suggests they may be “receivers” rather than “broadcasters” of deregulations. In (Gil Perez et al., 2024), we listed the main properties of *EPHA10.* It is a known oncogene. Its m6A-methylation is regulated by RBM15 B. It is known to activate the ERK/AKT pathway (Hu et al., 2025) and probably participate in immune evasion (Wang et al., 2024; Zhao et al., 2022).
- There are 1,605 orphan nodes (in-degree = 0) – these can only be spontaneously activated–and 513 child-less nodes (out-degree = 0) – these are endpoints of cascades.

### 3.2 Projecting samples onto the T-GDN reveals spontaneous vs. cascade activation

One of the most powerful features of our GDN framework is the ability to project each individual tumor sample onto the network and identify which deregulations are likely to have arisen spontaneously (i.e., without a deregulated parent in that sample) versus through cascade propagation.

**FIGURE 2:**
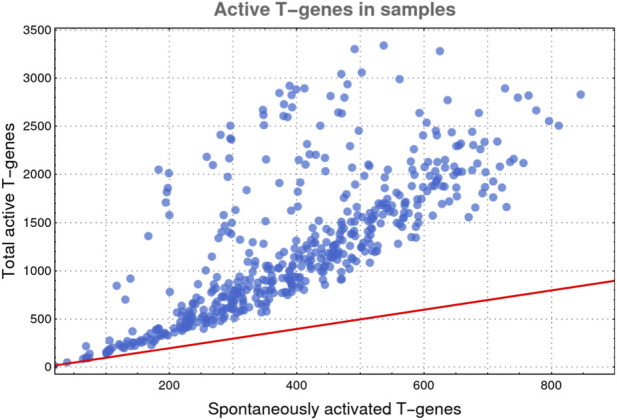
Spontaneous vs. cascade activation in PRAD tumors. Each point is a tumor sample. Y-axis: total number of active T-genes. X-axis: number of spontaneously activated genes (no active parent). Red line: total = spontaneous (no cascades). Early tumors (lower left) lie near the line; advanced tumors (upper right) lie far above, indicating wide cascades.

Figure 2 plots for each PRAD tumor sample the total number of deregulated (active) T-genes against the number of spontaneously activated genes in that sample. The red diagonal line represents the scenario where every deregulation is spontaneous (no cascades). We observe a clear gradient:

- Early-stage tumors (lower left quadrant, total deregulations <200) lie close to the diagonal: the majority of their active T-genes are spontaneous, with only a few cascade steps. This is consistent with the idea that newly formed tumors have not yet undergone sufficiently long evolution to develop long deregulation cascades.
- Advanced tumors (upper right quadrant, total deregulations >1,000) lie far above the diagonal: they have many more active genes than spontaneous ones, indicating that extensive cascade propagation has occurred. In some samples, the number of cascade-activated genes exceeds 2000.

This result provides direct, sample-level evidence that tumor progression is driven by the spread of deregulation through the GDN, and that the degree of cascade involvement correlates with the total number of active T-genes–which in turn was previously shown to correlate with the distance from the normal attractor (Gil Perez et al., 2024). A statistical analysis of the correlation between total active T-genes and the fraction of cascade-activated genes yields a Spearman’s ρ of 0.78 (p < 6 × 10^−103^), confirming the strong relationship.

Let us note that the degree of tumor progression, measured in terms of the distance to the center of the normal attractor, shows a poor correlation with tumor stage in PRAD (Gonzalez et al., 2021b). This is probably related to the heterogeneity of the prostate tumor. In less heterogeneous tumors the correlation between stage and distance is stronger (see for example Figure 5 in (Gonzalez et al., 2023)).

### 3.3 Paired normal-tumor samples: visualizing the transition

**FIGURE 3:**
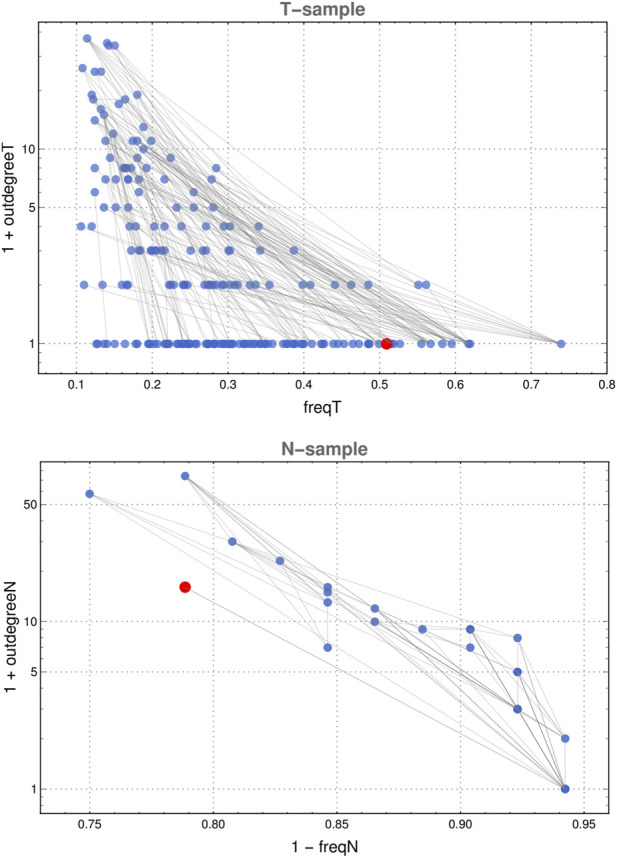
Paired normal (bottom) and tumor (top) samples from the same patient. Top: active T-genes, with edges from the T-GDN. Bottom: active N-genes, with edges from the N-GDN. Red node: *DNAJB4*, an NT-gene active in both states. The tumor is early (only 199 active T-genes), yet cascades are already visible. Deactivation pressure from already-deactivated nodes (not shown) also affects active N-genes.

Figure 3 shows a matched pair (same patient) of a normal sample and a tumor sample close to the transition point. Due to the relatively small number of active genes in each case, the cascades in the T- and N-GDNs may be easily visualized.

The tumor sample is in an early-stage (199 active T-genes) (Gil Perez et al., 2024). In the top panel, we plot all active T-genes, with edges from the T-GDN. Many genes do not have parents in the diagram, they are likely to be spontaneously activated. Deregulation cascades are also apparent in the Figure. The structure is highly branched - not a simple linear chain. One gene, *DNAJB4* (an NT-gene), is active in both the normal sample (as an N-gene) and the tumor sample (as a T-gene). This is a concrete example of an NT-gene switching roles during the transition.

The normal sample (bottom panel) has only 32 active N-genes remaining (27 block genes). This is an advanced stage of somatic evolution. The fact that only a handful of N-genes are still active in this normal sample suggests that the loss of N-gene protection is nearly complete (Gil Perez et al., 2024).

We note that the active N-genes in this sample include nodes with relatively high-out-degree, indicating that they are “keystone” genes whose deactivation would likely trigger widespread loss of the normal state.

Let us stress that on the active N-nodes there is also the deactivation pressure exerted by the inactive nodes, not represented in Figure 3 for the sake of simplicity.

### 3.4 T-GDNs across five cancer types: comparison and emerging patterns

We extended the construction of T-GDNs to four additional cancer types: HNSC, LUAD, UCEC, and LUSC. These were selected because they span a wide range of distances between the normal and tumor attractors, as previously characterized in (Gonzalez et al., 2021a). As shown in (Gil et al., 2025), the distance between the normal and tumor attractors seems to be an important characteristic of tumors, not used in the clinical practice so far.

Table 1 summarizes the global properties of the T-GDNs for all five types.

Several trends are noteworthy:

- The number of T-genes increases with the inter-attractor distance, from ∼6,000 in PRAD (smallest distance) to ∼18,600 in LUSC (largest distance). This suggests that in cancers that are more transcriptionally distinct from the normal tissue, more genes become exclusive markers of the tumor state.
- Sparsity remains consistently low (0.2% - 0.3%), indicating that regardless of cancer type, deregulation propagates through a small fraction of all possible gene pairs. The networks are not dense random graphs.
- Maximum deregulation frequency rises from 0.74 (PRAD) to 0.998 (LUSC). In LUSC and UCEC, we find almost perfect T-markers - NT-genes that are deregulated almost in every single tumor sample and never in normal samples. These are excellent diagnostic candidates, as previously reported (Gil et al., 2025).
- Mean in-degree/out-degree varies across types, with LUSC showing the highest values (mean degree ∼56), reflecting a more interconnected network. This may correspond to a higher degree of transcriptional reorganization.
- Network diameter (longest shortest path) is consistently similar in all cases, around 2.4.

The results indicate that while the overall architecture of GDNs is conserved (sparse, DAG, low-frequency/high-out-degree nodes, high-frequency/high-in-degree nodes), the quantitative parameters are cancer-type specific, likely reflecting different underlying mutational and epigenetic landscapes.

### 3.5 The N-GDN in PRAD: compressed nodes and discrete steps

The N-GDN in PRAD has 1097 N-genes, but after merging blocks of identical profiles we obtain only 492 representative nodes. The largest block contains 216 genes (see Table 1). Although we can not completely discard the effect of sample size, we interpret this as evidence for coordinated, multi-gene deactivation events during somatic evolution. In the multi-step model of cancer (Frank, 2007), each “step” is typically associated with a genetic or epigenetic alteration. Here we suggest that a single such alteration may lead to the simultaneous loss of expression of dozens or even hundreds of N-genes, possibly through the inactivation of a master transcriptional regulator or the condensation of chromatin.

**FIGURE 4:**
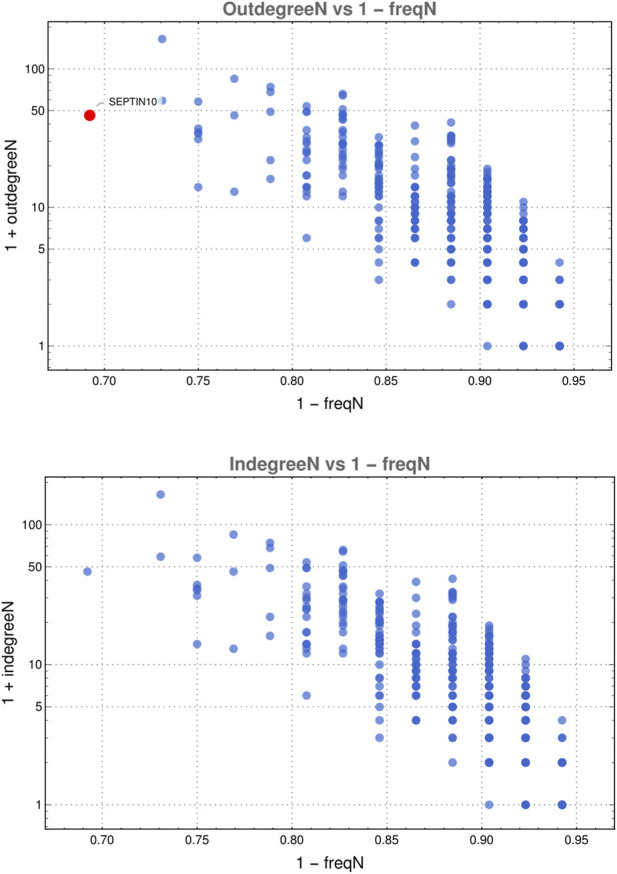
N-GDN of PRAD (after merging blocks). *SEPTIN10* (lowest deactivation frequency, 0.69) has a high out-degree (45). Each node may represent dozens of genes (largest block: 216 genes).

Figure 4 shows the degree distributions of nodes in N-GDN (compressed) for PRAD. *SEPTIN10* (an NT-gene) has the lowest deregulation frequency (0.69), meaning it is active (i.e., e = −1) in 31% of normal samples. It also has the highest out-degree (45). This makes *SEPTIN10* a key anchor of the normal state: its deactivation likely triggers a cascade that deactivates many other N-genes.

Note that *SEPTIN10* is simultaneously the gene with strongest deactivation capacity and with the highest relevance for normal tissue identity. In (Gil Perez et al., 2024), its known characteristics were listed, as for example, that it ensures accurate cell division (through its interaction with *BUB3*) and facilitates the death of cells with damaged microtubules (Xu et al., 2012). *SEPTIN10* acts as a dual caretaker and gatekeeper. Its protective function is likely dependent on the specific protein environment of a healthy prostate cell. If that environment changes - for example, if a tumor does form - *SEPTIN10*’s role could be subverted to support cancer progression (Poüs et al., 2016). This is consistent with its NT character.

Other notable nodes in the N-GDN include androgen-regulated genes (e.g., *PARM1*, which is also an NT-gene) and stress-response genes (e.g., *HSPA1L* block of 4 genes) (Gil Perez et al., 2024).

### 3.6 Comparison of N-GDNs across cancer types and contrast with T-GDNs

While the T-GDN properties vary markedly across cancer types (Section 3.4, Table 1), the N-GDNs show a remarkable quantitative similarity regardless of the tissue of origin. This is consistent with the hypothesis that the number of N-genes–and the overall architecture of the normal-state regulatory network–is an intrinsic property of the homeostatic state, rather than a cancer-specific adaptation.

#### 3.6.1 Similarities across N-GDNs

For the five cancer types analysed (PRAD, HNSC, LUAD, UCEC, LUSC), the number of N-genes remains within a relatively narrow range (492–1,372), despite the large variation in T-gene counts (6,138 – 18,596). After merging blocks of identical expression profiles, the number of representative nodes in the N-GDN is between 492 and 1,228 (see Table 1). The sparsity of the N-GDN is consistently higher (0.01 - 0.02) than that of the T-GDN (0.002 - 0.003), meaning that N-genes are more densely interconnected.

The phenomenon of gene blocks–sets of N-genes with identical deregulation profiles–is observed in all cancer types. PRAD exhibits the largest block (216 genes), and the two lung cancer localizations the lowest counts of number and sizes of blocks. Subsampling analysis in PRAD (Supplementary Table S3), although not definitive, seems to confirm that these blocks are not sampling artifacts; they likely represent coordinated transcriptional modules that are lost in a single step during somatic evolution. This suggests that the multi-step deactivation of N-genes occurs in a tissue-specific but quantitatively similar manner.

### 3.7 Contrast with T-GDNs

The differences between N-GDNs and T-GDNs are striking and biologically informative:

- Number of nodes: T-GDNs are 5- to 15-fold larger than their corresponding N-GDNs (e.g., PRAD: 6,138 vs. 1,097; LUSC: 18,596 vs. 1,269). This reflects the higher transcriptional entropy of tumors (Mesa-Rodríguez et al., 2022; Gonzalez et al., 2022): many more genes can adopt a tumor-specific expression state than can maintain a normal-specific state.
- Sparsity and connectivity: N-GDNs are about one order of magnitude denser (sparsity ∼0.01–0.02) than T-GDNs (∼0.002). This suggests that the normal state relies on a more tightly knit network, where deactivation of one N-gene more directly facilitates deactivation of others. In contrast, the tumor network is more sparse, possibly because tumor progression can follow many alternative routes.
- Block structure: Large blocks are common in N-GDNs but rare in T-GDNs. This supports the view that somatic evolution proceeds via discrete, coordinated steps, whereas clonal evolution in tumors is more gradual and heterogeneous, with fewer perfectly correlated expression changes.

### 3.8 Possible implications for carcinogenesis

The consistent size and structure of N-GDNs across distinct tissues indicate that the protective machinery against tumor formation is quantitatively comparable from one tissue to another, even though the specific gene identities differ. In contrast, the T-GDN expands and reconfigures depending on how far the tumor expression profile has diverged from the normal attractor (Gonzalez et al., 2021a; Gonzalez et al., 2023). Cancers with larger attractor distance (e.g., LUSC) have more T-genes and more interconnected T-GDNs, suggesting that they have undergone more extensive transcriptional reprogramming. This is consistent with clinical observations: LUSC is generally more aggressive and has a poorer prognosis than PRAD, which often remains indolent for many years.

### 3.9 Simulating interventions: why targeting a perfect panel may fail

We simulated two interventions on a late-stage PRAD tumor sample (2,640 active T-genes; this sample is from the upper right of Figure 2):

1. Knockdown of *EPHA10* alone (the most frequent T-gene, frequency 0.74, in-degree 185).
2. Knockdown of all 8 perfect panel T-genes (according to (Gil Perez et al., 2024), at least one gene of this set is active in every tumor sample: *EPHA10*, *UCN, TMEM86A, RPL26P23, RBM15B, NPY4R2, TEKT4*, and *ENSG00000271895*).

**FIGURE 5:**
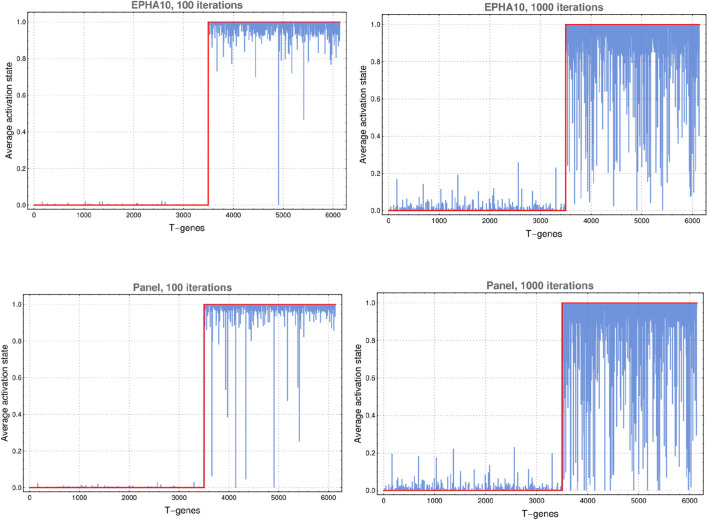
Simulation of forced deactivation in a late tumor (2,640 active T-genes). Top: deactivating *EPHA10* only. Bottom: deactivating all 8 perfect panel genes. Left: after 100 iterations; right: after 1,000 iterations. In both cases, the tumor recovers after 1,000 iterations–escape is due to incomplete reachability of the reversed network.

In both simulations, we allowed the direct dynamics (activation of T-genes along forward edges) to continue at a rate of p_direct_ = 0.1 per time step, while the forced deactivation propagated along reverse edges with p_force_ = 0.5. As mentioned in Section 2.6, the direct process is included to model the tumor’s attempt to recover. We ran the simulation for 100 and 1,000 iterations (pseudo-time). Results are shown in Figure 5.

After 100 iterations, forced deactivation of *EPHA10* alone reduces the average activation state (fraction of active genes) for many genes, particularly those upstream of *EPHA10*. However, after 1,000 iterations, the tumor “escapes”: many genes that were initially inactive become activated, and the overall activation profile recovers. The same pattern, even more pronounced, is observed when we knock down the entire 8-gene panel.

Why does targeting the entire perfect panel fail? The reason lies in the topology of the T-GDN. The reverse cascade starting from the panel genes does not reach the whole network. There exists a set of genes (about 30% of all T-genes) that are not reachable by reverse walks from any panel gene. These genes can become spontaneously activated (or activated through other paths) and then drive the tumor recovery. In other words, a perfect diagnostic panel (in which at least one of the genes is active in every tumor) is not necessarily a perfect therapeutic panel (genes whose deactivation would collapse the entire network). This is a crucial insight: therapies should target genes that simultaneously reach the highest fraction of tumors and which reverse cascade reaches the highest fraction of the network. This prediction is further developed in the companion paper (Gil et al., 2026).

### 3.10 Preliminary experimental check: *POM121* knockdown

**FIGURE 6:**
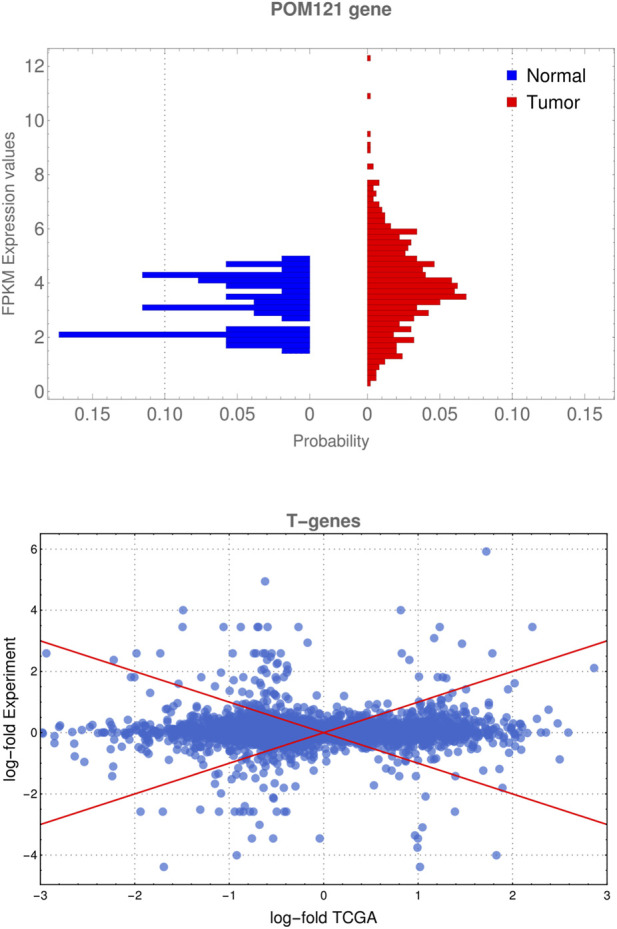
Preliminary experimental check with *POM121* knockdown. Top: TCGA histograms of *POM121* (tumor-above gene). Bottom: Comparison between log-fold change of expressions for T-genes in the experiment and in the TCGA data for the X22RV1 cell line. This comparison is used to define activated or deactivated genes.

We used the reported experiment on the knockdown of *POM121* in two prostate cancer cell lines (Kirthika et al., 2025) as a qualitative test of our cascade directionality prediction. *POM121* is a tumor-above gene with frequency 0.248 (intermediate, between the minimum 0.1 and the maximum 0.74). Paired histograms are shown in Figure 6, top panel. The mean value defined in Section 2.6, Δ_TCGA_ is.

−0.254 (normal vs tumor). The bottom panel of the Figure illustrates our approximate scheme for identifying activation and deactivation events. It also points out the source of errors: our T-genes are not defined in terms of Δ_TCGA_, which may take values near 0. However, a value of Δ_exp_ near zero is not statistically reliable. For this reason, we restrict the analysis to genes with |Δ_exp_| > |Δ_TCGA_| > 0.1.

According to our T-GDN, forced deactivation of *POM121* should preferentially deactivate genes with equal or lower frequency (because those are the only genes that can lie along the reverse propagation pathway). Table 2 and Figure 6 show the results for the X22RV1 cell line. The PC3 line gave very similar numbers.

- Among the 4119 T-genes with frequency <0.248, we observed 172 deactivated (fraction 0.04).
- Among the 1374 T-genes with frequency >0.248, only 28 were deactivated (fraction 0.02).

**TABLE 2:** Numbers of activated/deactivated genes in the *POM121* knockdown experiment (X22RV1 and DU145 cells).

X22RV1 cell line		
T-genes	Activated	Deactivated
FreqT < FreqT (POM121)	215/4,119	172/4,119
FreqT > FreqT (POM121)	37/1,374	28/1,374
N-genes		
​	62/1,009	40/1,009

DU145 cell line		
T-genes	Activated	Deactivated
FreqT < FreqT (POM121)	400/4,119	553/4,119
FreqT > FreqT (POM121)	56/1,374	98/1,374
N-genes		
​	87/1,009	75/1,009

T-genes are split by frequency relative to *POM121* (0.248). The fraction deactivated is much higher in the lower-frequency group (p ∼ 10^−5^). N-genes show no consistent pattern.

The association between frequency interval and deactivation status is highly significant (Fisher exact test, p ∼ 4 × 10^−5^).

Moreover, we examined whether the deactivated low-frequency genes were enriched with those directly reachable from *POM121* by reverse edges. Out of 172 deactivated genes with frequency <0.248, 8 were in the reverse reachable set (which contains 133 genes total). This is a 6% hit rate, compared to a background of 4% (172/4,119). An exact Fisher test leads to a p-value of 0.09. The 8 deactivated genes in the cascade are: *BLTP2* (linked to a cancer-associated expression program, particularly in prostate cancer) *POLR3B* (broad roles in gene expression (transcription) and related regulatory pathways)*, GOSR1, MTCL2* (role in microtubule organization in proliferative and specialized cellular contexts)*, TMEM123* (cell surface receptor mediating cell death)*, BACH1* (transcriptional regulator with roles in gene expression control and DNA repair)*, MAML1* (transcriptional coactivator with relevance in prostate cancer, glioma susceptibility 1, and soft tissue sarcoma)*, ZNF792* (*KRAB* zinc-finger transcription factor). This suggests that our GDN correctly identifies some genuine upstream targets.

For N-genes (which should not be active in a tumor), we observed 40 deactivations (noise level) and 62 activations. The activation events are slightly above noise, possibly due to NT-gene cross-talk (some NT-genes could be T-deactivated in the T-GDN, indirectly affecting other N-genes).

We conservatively conclude that the T-GDN predictions are qualitatively confirmed, but a more definitive validation would require a larger-scale perturbation study with multiple knockdowns and single-cell readouts.

## 4 Discussion

### 4.1 Summary of contributions

We have introduced Gene Deregulation Networks (GDNs) as a scalable, data-driven, and annotation-free framework for modeling cancer progression. Two practical advantages distinguish GDNs from all other GRN inference methods:

- Sample-specific cascade analysis: By projecting a sample’s deregulation profile onto the GDN, one can immediately see which genes were spontaneously activated and how the deregulation propagated. This provides a personalized “cascade history” that could, in principle, be used to stratify patients by their tumor’s evolutionary stage.
- Trivial dynamics: Spontaneous evolution follows the GDN edges; forced interventions follow the reversed network. No complex parameter fitting is required–the network topology alone suffices to simulate progression or response to therapy. This simplicity allows rapid *in silico* screening of potential therapeutic targets.

### 4.2 Relationship to the multi-step model of cancer

The presence of large gene blocks (e.g., 216 genes with identical expression profiles) in the N-GDN of PRAD strongly suggests that somatic evolution does not occur gene-by-gene but in discrete, coordinated steps. Large blocks may correspond to “hits” in the classic multi-step model (Frank, 2007). This is an important refinement: rather than thinking of sequential mutations in individual oncogenes or tumor suppressors, our results indicate that each step may involve the simultaneous deregulation of dozens or hundreds of genes, likely through epigenetic mechanisms or master transcription factor switches. The T-GDN, by contrast, shows gradual, branching cascades–consistent with clonal evolution where many small steps accumulate.

### 4.3 Limitations and future work

We acknowledged the limitations of our framework and have addressed them throughout the manuscript. For clarity, we summarize them here:

- Bulk RNA-Seq: The data we use average over many cell types (malignant, stromal, immune). This may create spurious correlations and cannot resolve intra-tumor heterogeneity. Single-cell RNA-seq will be required to confirm cascade directionality at the cellular level. Extending the GDN framework to single-cell data is a task for the near future, but this is non-trivial due to dropout and sparsity.
- Cross-sectional design: We use the ergodic principle to infer dynamics from static snapshots. This assumes that the cohort spans all stages of progression and that different patients’ samples can be aligned along a common trajectory. Longitudinal or time-series data (e.g., from organoid models) would provide stronger evidence regarding the inferred dynamics.
- No gold standard: We cannot compute standard metrics (AUROC, precision-recall) because no validated human cancer network exists. Our validation therefore relies on internal consistency, stability across subsamples, and qualitative agreement with perturbation experiments. The *POM121* knockdown is a first step; more systematic perturbations (e.g., CRISPR screens targeting dozens of genes) are needed.
- Causal interpretation: Our links represent *prima facie* relations of causal sufficiency, refined through several filtering stages (see Sec. 2.3; S1), not necessarily true causation. We use the term “causal” in the probabilistic sense of Suppes and Reichenbach (Suppes, 1970; Reichenbach, 1971; Cartwright, 1979), not as proven biological causality, which should be ultimately established in intervention experiments. This distinction should be kept in mind when interpreting the inferred GDNs.

Despite these limitations, GDNs provide a practical tool for hypothesis generation and for designing intervention strategies. The failure of the 8-gene panel to eliminate the tumor *in silico* (Section 3.7) suggests that the topological structure of GDNs may contain relevant information for the design of RNA therapies. In the companion paper (Gil et al., 2026), we use the GDN framework to predict specific RNA therapeutic interventions that could partially reverse the tumor phenotype by targeting NT-genes that bridge the N- and T-networks. These are testable predictions.

### 4.4 Conclusion

We have shown that Gene Deregulation Networks can be reliably inferred from TCGA bulk data, that they recapitulate known biological patterns (e.g., *EPHA10* as a hub, *SEPTIN10* as a key N-gene anchor), and that they enable two unique functionalities: personalized cascade analysis and topology-driven dynamics simulation. The separation into N- and T-GDNs provides a natural decomposition of carcinogenesis into two distinct phases (somatic evolution and clonal evolution). Future work will extend the framework to single-cell data, integrate it with perturbation screens to validate causal predictions, and apply it to the rational design of RNA-based therapies. As detailed in (Gil et al., 2026), the GDN framework generates specific, testable predictions for gene knockdown and overexpression experiments, and we hope that this will stimulate further experimental testing.

## Data Availability

All data used in this study are publicly available through the TCGA Research Network (https://www.cancer.gov/tcga). The complete source code of CChains, along with example input files and a README, is available at https://github.com/gabriel-gil/CChains (last commit on June 8th, 2026). A companion repository for the GDN framework, available at https://github.com/fcastelm/GDN, provides analysis code together with CChains example inputs and outputs required to construct, inspect, and reproduce the GDNs presented in this work (last commit on June 4th, 2026). The binary deregulation matrix used as input by CChains is generated using the latest version of our GenePan code, available at https://github.com/gabriel-gil/GenePan (last commit on June 3rd, 2026).
