# Supplementary material for: N-Gene and T-Gene deregulation networks: a data-driven causal framework for the analysis of gene interventions in cancer
Source: Front Syst Biol. 2026 Jul 2;6:1861211. doi: 10.3389/fsysb.2026.1861211 (PMC13372580; doi:10.3389/fsysb.2026.1861211)
Supplement: Supplementary file 1 [file DataSheet1.PDF]

N-Gene and T-Gene Deregulation Networks: A data-driven causal framework for the analysis of gene interventions in cancer

Frank Castel[1], Roberto Herrero[1], Jean Pierre Gómez[2], Gabriel Gil[1], and Augusto Gonzalez[1]*

Supplementary Material

S0. Examples of N-genes, T-genes, NT-genes and O-genes

S0.1 Continuous-expression toy example and gene-class identification

This example follows the same computational workflow as the article. We start from a continuous expression matrix containing ten toy genes measured in 10 normal samples and 25 tumor samples. The continuous matrix is split into normal and tumor columns only for readability:

Table S0.1. Continuous expression matrix for the normal samples (FPKM).

gene	n1	n2	n3	n4	n5	n6	n7	n8	n9	n10
g1	0.8	1.4	2.2	3.2	4.1	4.9	5.5	6.1	6.8	7.3
g2	1.8	2.4	2.9	3.5	4.2	4.8	5.3	5.9	6.4	7.0
g3	2.4	3.1	3.7	4.9	5.6	6.4	7.1	7.9	8.4	9.0
g4	0.7	1.2	1.7	2.4	2.9	3.5	4.0	4.5	5.0	5.2
g5	3.6	4.6	5.4	6.4	7.5	8.7	9.5	10.4	11.1	11.6
g6	9.8	10.6	11.3	12.1	8.6	7.5	13.4	14.0	6.8	12.8
g7	1.1	0.8	0.6	1.3	2.1	2.6	0.7	1.5	3.1	0.4
g8	2.5	3.5	4.8	5.6	6.3	7.0	7.7	8.4	5.1	6.8
g9	1.1	2.0	3.1	3.9	4.6	5.4	6.2	7.0	7.9	8.5
g10	3.2	4.1	4.9	5.8	6.6	7.4	8.2	9.1	6.0	7.2

Table S0.2. Continuous expression matrix for the tumor samples (FPKM).

gene	s1	s2	s3	s4	s5	s6	s7	s8	s9	s10	s11	s12	s13	s14	s15	s16	s17	s18	s19	s20	s21	s22	s23	s24	s25
g1	1.0	3.1	0.7	5.0	14.4	10.7	1.3	5.8	14.3	4.0	15.0	12.0	1.4	5.5	2.0	11.5	2.3	11.8	1.8	9.5	16.4	0.8	9.7	4.9	6.7
g2	2.9	4.7	0.0	5.2	0.8	0.3	4.2	3.5	0.6	5.5	0.9	2.5	2.0	7.2	0.7	0.3	1.0	0.2	0.4	0.7	2.8	1.0	0.6	0.5	6.5
g3	6.5	6.3	13.7	0.5	16.8	13.3	1.8	2.8	0.6	12.4	12.6	17.4	2.8	0.3	11.6	0.7	13.6	0.8	0.4	15.3	2.5	7.9	18.4	15.6	2.5
g4	3.6	9.5	2.0	3.0	7.3	11.5	2.8	10.6	8.0	2.7	1.5	6.8	1.1	0.5	6.5	10.1	11.7	10.6	8.2	12.3	4.5	4.1	11.7	11.0	3.9
g5	2.4	19.5	10.7	18.6	14.5	19.0	5.3	7.8	19.4	4.7	18.0	21.2	2.2	5.6	16.1	0.5	21.4	20.8	18.7	20.7	14.2	10.2	1.1	1.5	19.9
g6	1.2	2.1	3.0	4.7	5.8	7.9	8.4	2.7	6.5	3.9	5.2	1.6	4.1	7.0	8.1	2.4	3.6	5.7	6.9	4.8	7.6	2.9	3.3	5.9	6.2
g7	2.0	2.8	3.4	4.1	5.9	6.7	7.5	2.3	3.1	4.8	5.4	6.1	7.0	2.7	3.6	4.4	5.2	6.5	7.2	3.9	4.7	5.8	6.9	2.5	3.3
g8	2.5	3.8	4.4	5.2	6.0	6.7	7.5	8.1	8.0	4.9	5.7	6.4	7.1	3.3	8.2	8.4	5.5	6.2	7.8	4.1	3.6	8.1	6.9	5.0	7.3
g9	0.9	1.4	2.1	2.8	3.2	4.0	4.7	5.3	6.1	6.8	7.5	8.2	3.6	4.4	5.8	6.5	7.1	2.5	1.8	8.7	5.0	4.2	6.9	3.0	7.8
g10	2.7	3.6	4.5	5.4	6.2	7.0	7.8	8.6	9.4	5.2	6.1	7.3	8.1	3.9	4.7	9.0	6.5	7.6	8.4	5.7	4.2	3.3	6.9	8.8	7.1

From these continuous values, genes are classified as T-genes, N-genes, or O-genes according to whether a class-exclusive expression interval can be defined for tumor samples, normal samples, or neither (see Tab. S0.3). N- and T-genes are further classified as Only-X-above, Only-X-below, and Only-X-outside according to whether their active state lies above or below an expression threshold, or outside an expression interval. Notice that the activation frequencies in Table S0.3 are all above the minimum frequency thresholds of 0.1 for T-genes and 0.05 for N-genes.

Table S0.3. Gene classification from the continuous expression matrix.

gene	classification	family	lower threshold	upper threshold	rule	activation frequency
g1	T-gene	Only-T-above	8.4		FPKM > 8.4	10/25
g2	T-gene	Only-T-below	1.4		FPKM < 1.4	14/25
g3	T-gene	Only-T-outside	2.1	10.3	FPKM < 2.1 or FPKM > 10.3	18/25
g4	T-gene	Only-T-above	5.9		FPKM > 5.9	14/25
g5	T-gene	Only-T-outside	3.0	12.9	FPKM < 3.0 or FPKM > 12.9	19/25
g6	N-gene	Only-N-above	9.1		FPKM > 9.1	7/10
g7	N-gene	Only-N-below	1.8		FPKM < 1.8	7/10
g8	O-gene				no class-exclusive interval	
g9	O-gene				no class-exclusive interval	
g10	O-gene				no class-exclusive interval	

Continuous matrices from Table S0.1 and S0.2 are discretized into three categorical expression values for N- and T-genes following the classification and rules from Table S0.3: e = -1 iff the expression lies in a normal-exclusive interval, e = 1 iff the expression lies in a tumor-exclusive interval, and otherwise e = 0.

Table S0.4. Binary T-gene activity patterns from the T-exclusive rules.

gene	s1	s2	s3	s4	s5	s6	s7	s8	s9	s10	s11	s12	s13	s14	s15	s16	s17	s18	s19	s20	s21	s22	s23	s24	s25
g1	0	0	0	0	1	1	0	0	1	0	1	1	0	0	0	1	0	1	0	1	1	0	1	0	0
g2	0	0	1	0	1	1	0	0	1	0	1	0	0	0	1	1	1	1	1	1	0	1	1	1	0
g3	0	0	1	1	1	1	1	0	1	1	1	1	0	1	1	1	1	1	1	1	0	0	1	1	0
g4	0	1	0	0	1	1	0	1	1	0	0	1	0	0	1	1	1	1	1	1	0	0	1	1	0
g5	1	1	0	1	1	1	0	0	1	0	1	1	1	0	1	1	1	1	1	1	1	0	1	1	1

Table S0.5. Binary N-gene activity patterns from the N-exclusive rules.

gene	n1	n2	n3	n4	n5	n6	n7	n8	n9	n10
g6	1	1	1	1	0	0	1	1	0	1
g7	1	1	1	1	0	0	1	1	0	1

Figure S0.1. Visual identification of the T-gene examples. Each row corresponds to one toy gene. Equal-height spikes are positioned along the x-axis according to the FPKM expression of their corresponding sample; blue spikes denote normal samples and red spikes denote tumor samples. Horizontal orange segments indicate the T-exclusive interval(s), with vertical orange lines marking the threshold boundaries.

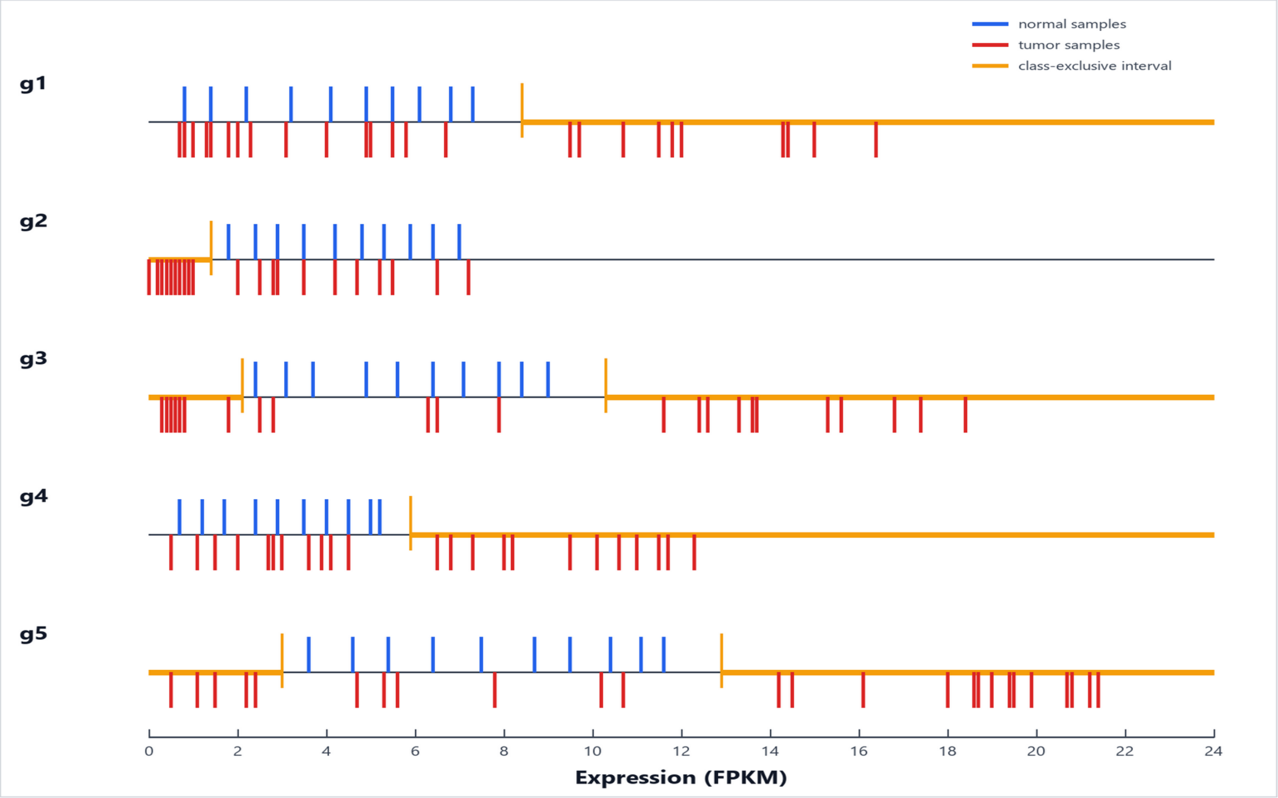

Figure S0.2. Visual identification of the N-gene examples. The same continuous-expression representation is used, but horizontal and vertical orange lines now indicate N-exclusive intervals.

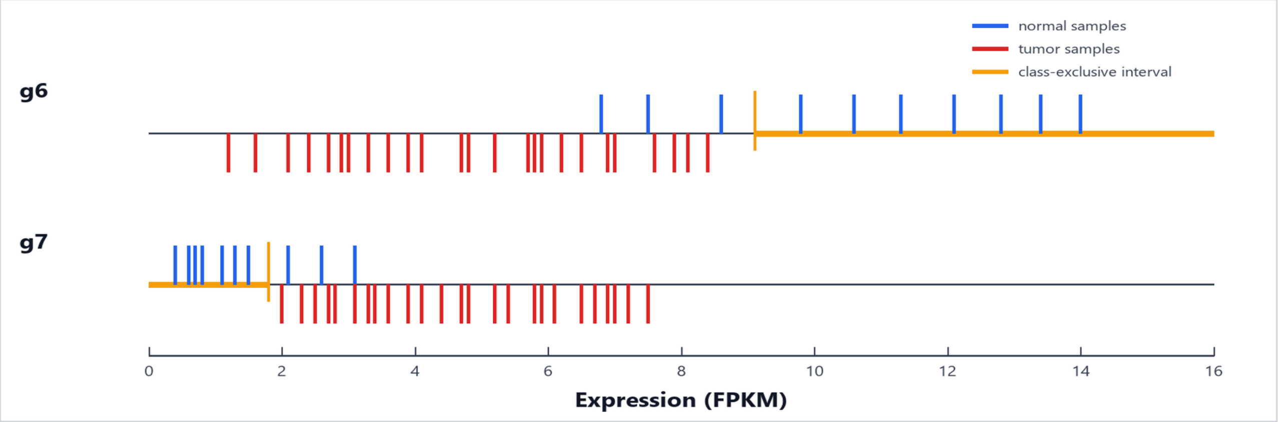

Figure S0.3. O-gene examples. Normal and tumor expression values overlap, preventing the identification of a significantly populated class-exclusive expression interval.

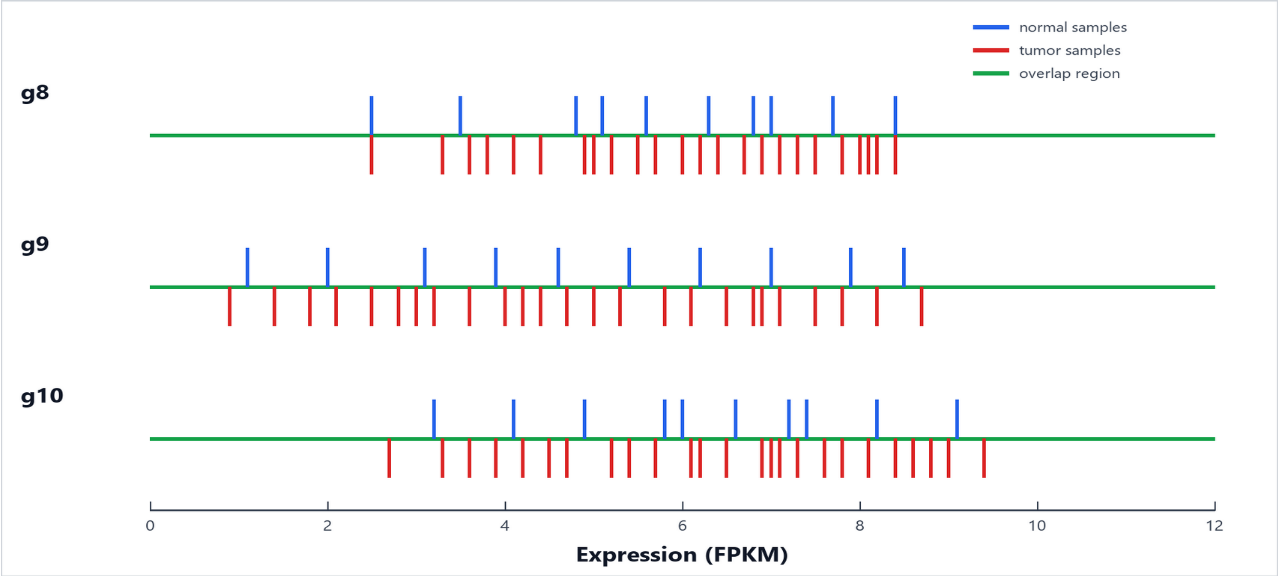

We borrowed from [15] real examples of genes in PRAD.

The *EPHA10* gene (a T-gene): In the Figure we show expression histograms in normal and tumor samples. FPKM expression intervals for normal and tumor samples are (0.04, 0.88) and (0.08, 5.25), respectively. Thus, there is an interval, (0.88, 5.25), that is exclusive to tumor samples. We discretize the expression and write e = 0 for normal and tumor samples in the common interval, e = 1 for tumor samples with expression above 0.88, and include the gene in the "tumor-above" group. Notice that e = 1 for this gene is an indication of a tumor, whereas e = 0 for all normal and for some tumor samples. When e = 1, we say the gene is T-activated.

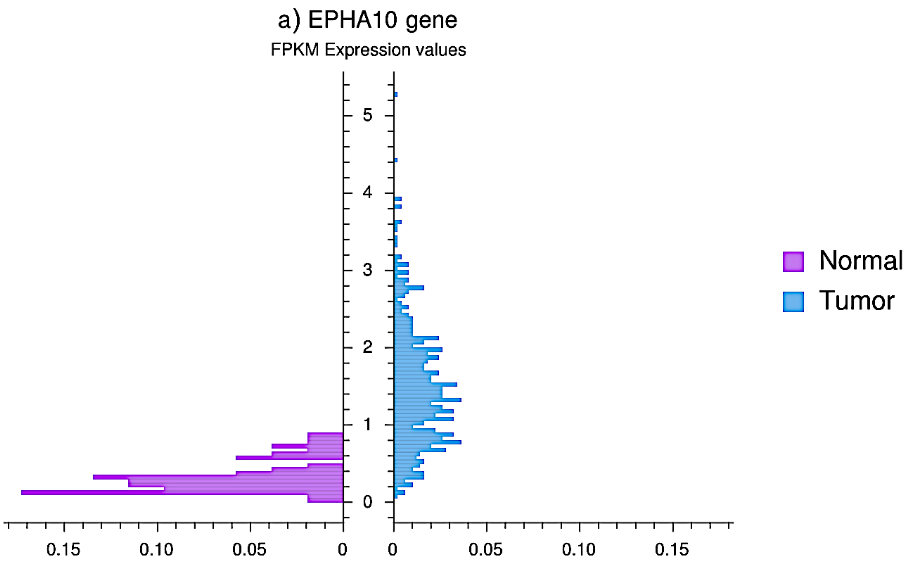

The *SEPTIN10* gene (an NT-gene): For N-genes there is an exclusive interval for normal samples. In this case, we set e = 0 for expression values of both tumor and normal samples in the common

interval, and e = −1 for normal samples in the normal-only interval. In the next Figure we show paired histograms for the *SEPTIN10* gene. As it is apparent, we may include this gene in the normal-above group. Expression values above 22.26 seem to be incompatible with the tumor state and are uniquely reached by normal samples.

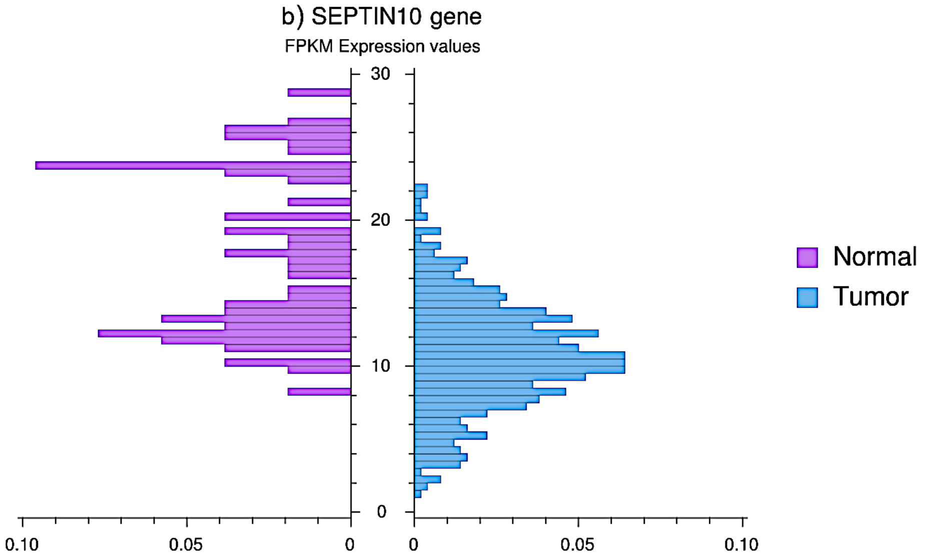

Notice that *SEPTIN10* also exhibits a tumor-only interval, in which the expression is below 8. Thus, it behaves as a normal-above gene, since its expression interval above 22.26 contains only normal samples, or as a tumor-below gene, since its expression interval below 8 contains only tumors. It is an NT-gene.

The *PCMTD1* gene (another T-gene): This example is illustrative of the non-differential character of our classification. The gene belongs to the tumor-outside group, that is, genes that are simultaneously tumor-above and tumor-below, in other words, there are expression intervals for tumor samples above and below the normal region. The common region for all normal and some tumor samples is sandwiched between the external intervals. The paired histograms are shown in the next Figure. This gene would not be identified in a differential expression approach, although it has been identified as a fusion gene in PRAD. Deregulation of this gene in expression data could be related to fusion events.

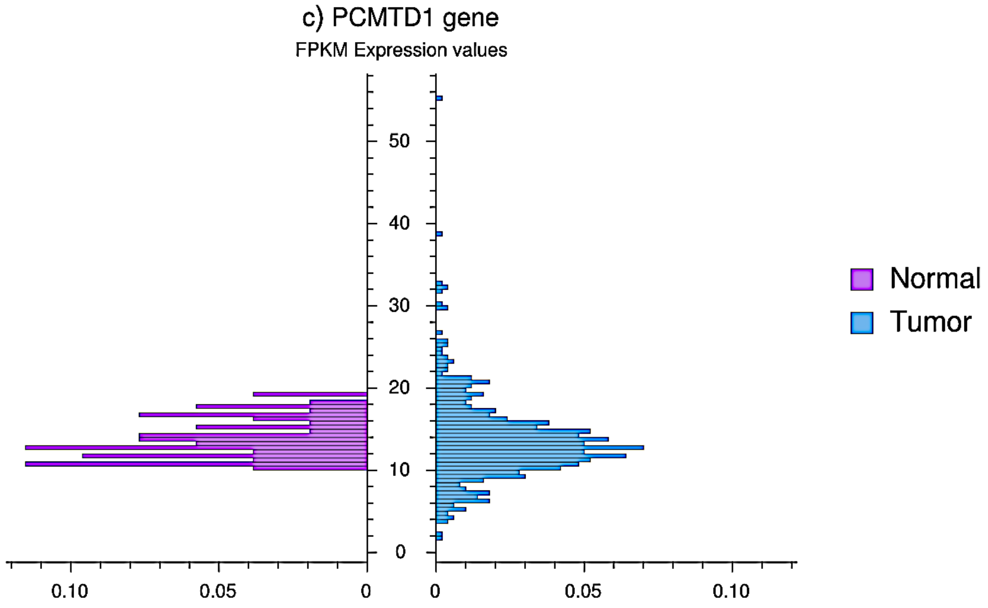

The *EHD4-AS1* gene (an O-gene): The *EHD4* Antisense RNA 1 is a good example of an O-gene. There are tumor samples outlayers in the high-expression and low-expression intervals, but the number of samples is not statistically significant. The activation state of this gene is always e = 0. Thus, it does not participate in the dynamics of normal samples, nor in the dynamics of tumor samples.

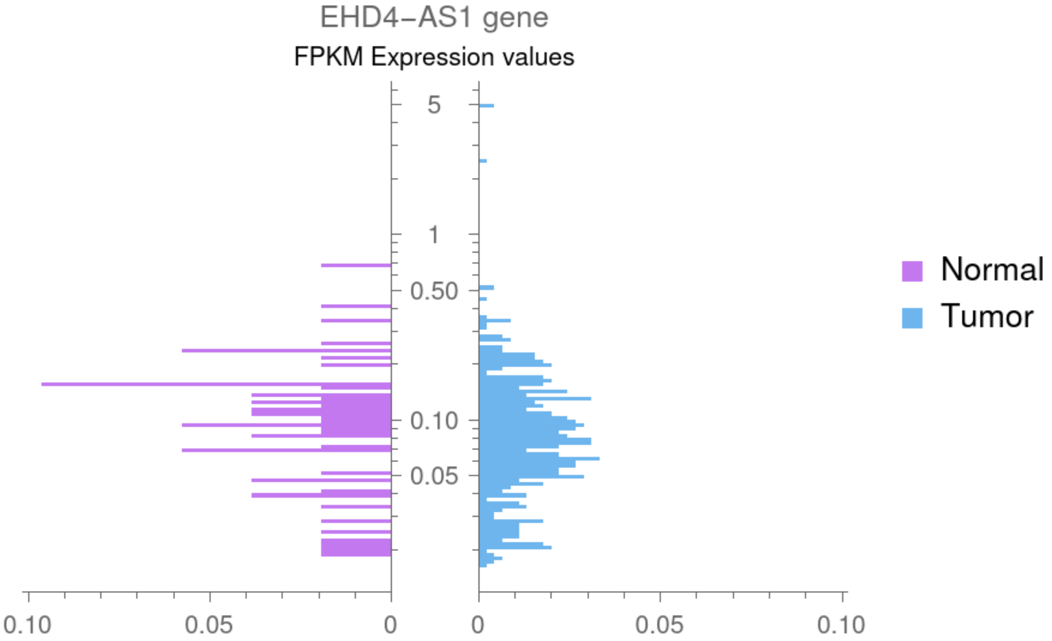

S1. Schematic pipeline of GDN construction and dynamics

We illustrate the framework and the algorithm for dynamical simulations in a toy example of T-GDN with 5 genes and 25 samples (extracted from last section).

S1.1 Deregulation matrix, i.e. the active T-genes in samples (g - genes, s - samples):

	s1	s2	s3	s4	s5	s6	s7	s8	s9	s10	s11	s12	s13	s14	s15	s16	s17	s18	s19	s20	s21	s22	s23	s24	s25
g1	0	0	0	0	1	1	0	0	1	0	1	1	0	0	0	1	0	1	0	1	1	0	1	0	0
g2	0	0	1	0	1	1	0	0	1	0	1	0	0	0	1	1	1	1	1	1	0	1	1	1	0
g3	0	0	1	1	1	1	1	0	1	1	1	1	0	1	1	1	1	1	1	1	0	0	1	1	0
g4	0	1	0	0	1	1	0	1	1	0	0	1	0	0	1	1	1	1	1	1	0	0	1	1	0
g5	1	1	0	1	1	1	0	0	1	0	1	1	1	0	1	1	1	1	1	1	1	0	1	1	1

S1.2 Frequencies:
$freq_1 = 10/25$, $freq_2 = 14/25$, $freq_3 = 18/25$, $freq_4 = 14/25$, $freq_5 = 19/25$

Coincidence frequencies $freq_{ij}$ (symmetric matrix):

	g1	g2	g3	g4	g5
g1		8/25	9/25	8/25	10/25
g2			13/25	11/25	12/25
g3				12/25	14/25
g4					13/25
g5					

S1.3 Loevinger coefficients $H_{ij} = (freq_{ij} - freq_i\, freq_j) / (freq_i\,(1 - freq_j))$:

	g1	g2	g3	g4	g5
g1	0	0.545	0.643	0.545	1
g2	0	0	0.745	0.513	0.405
g3	0	0	0	0	0.074
g4	0	0.513	0.490	0	0.702
g5	0	0	0	0	0

S1.4 Initial network. Edges with $H_{ij} > 0.5$ are preserved (node frequencies ordered along the x-axis):

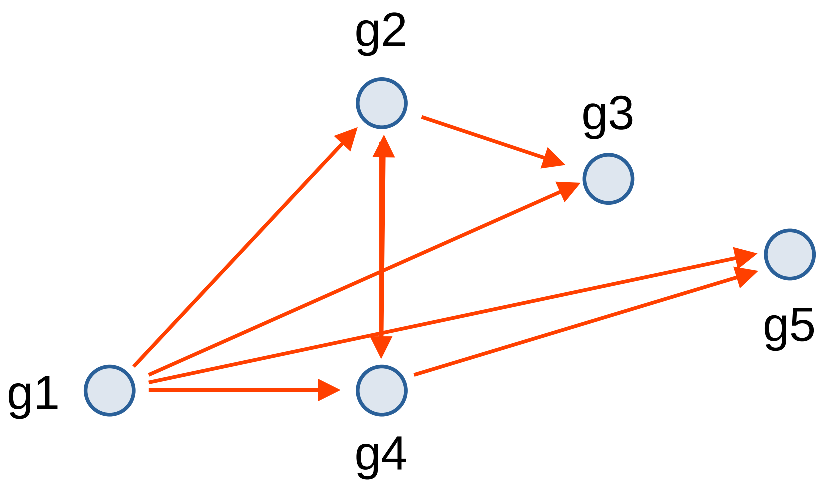

S1.5 Reichenbach simplification (common cause):

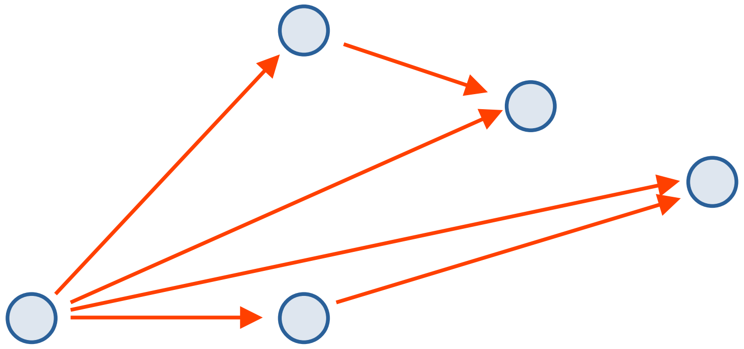

S1.6 Mokken simplification (transitivity):

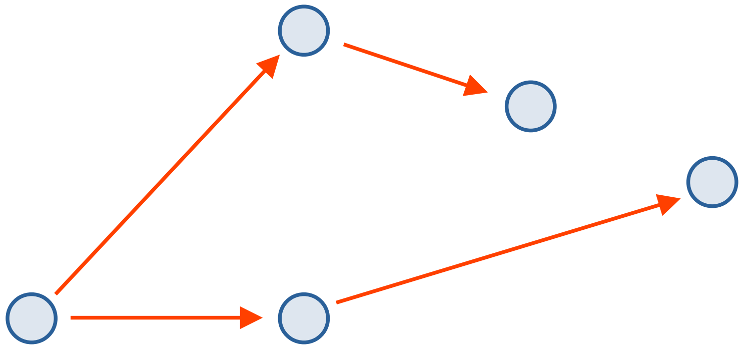

S1.7 Elimination of remaining double links: No double links between equal frequency nodes remained, thus the later is the final GDN.

S1.8 An hypothetical sample immersed in the network. It makes apparent the deregulation cascade g1 → g2 (active nodes in intense blue). Cascade analysis: g1 – spontaneously activated, g2 – activation through the cascade:

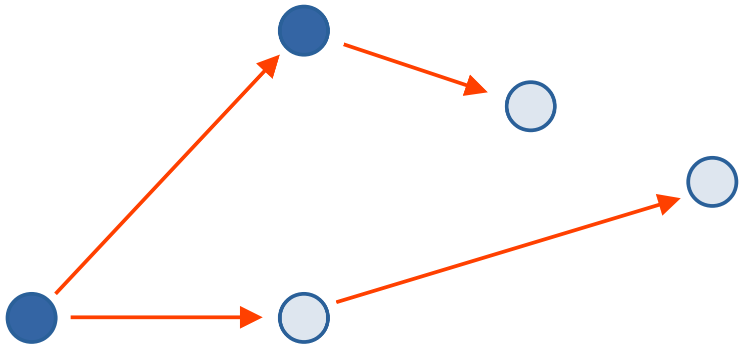

S1.9 A first possible step in dynamic evolution:

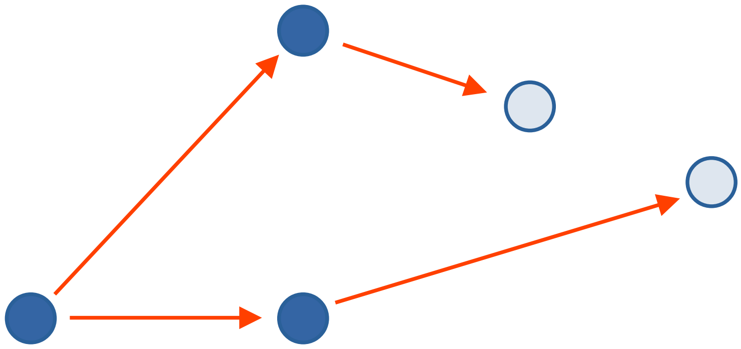

S1.10 Intervention. Initial network state (intervened node in red):

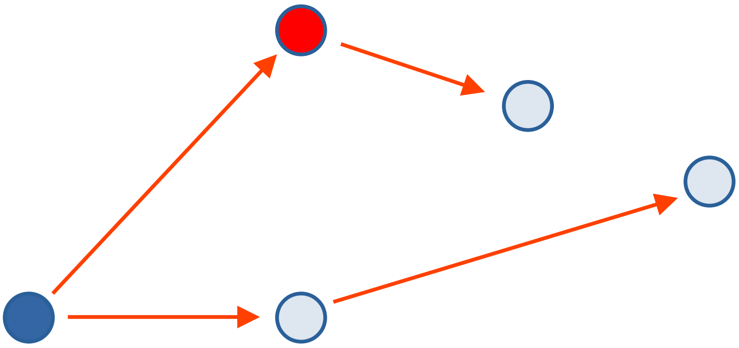

Tumor-free final state (one possible final state). The intervention evolves according to the transposed network:

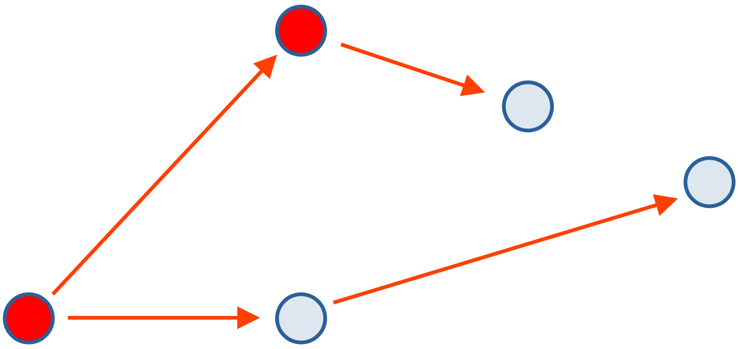

Escape (another possible final state). Node 4 activation preceded the deactivation of node 1:

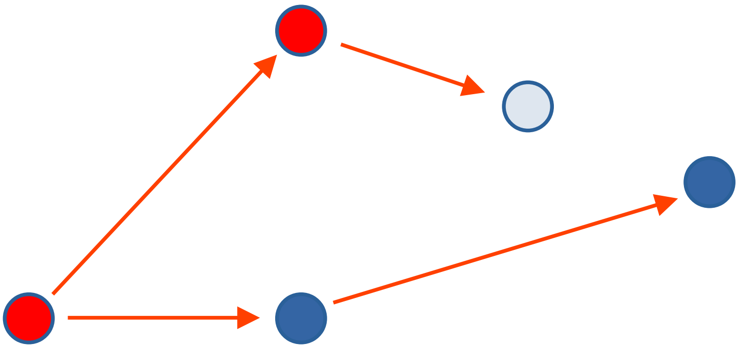

S2: Mathematical formulation of Reichenbach, Mokken and heuristic tests

S2.1 Reichenbach test

Some causal links ($i \rightarrow j$) discovered by our algorithm may be spurious, arising from non-causal statistical correlations between effects of a common cause k (i.e., $k \rightarrow i$ and $k \rightarrow j$) [5]. A Reichenbach-like test helps identify and remove such cases [21]. In practice, for any edge detected by the Loevinger criterion, we ask whether there exists a common cause gene such that

a) $H_{ij|k} = (v_{j|i\&k} - v_{j|k})/(1 - v_{j|k}) \leq H_0$, and

b) $H_{ij|\sim k} = (v_{j|i\&\sim k} - v_{j|\sim k})/(1 - v_{j|\sim k}) \leq H_0$.

In criteria a) and b), we compute conditional Loevinger coefficients by restricting the dataset to the deregulated and non-deregulated states of gene k, respectively, and then evaluating the co-occurrence frequency of deregulations at genes i and j, as well as the deregulation frequency of gene j, within each subset. Since $H_{ij} > H_0$ is interpreted as a prima facie measure of probabilistic causal sufficiency of i over j, the Reichenbach-like test evaluates whether this relation persists after fixing the deregulation status of a common-cause gene. If the conditional Loevinger coefficient falls below threshold in both the deregulated and non-deregulated cases of the common-cause gene, then the apparent causal sufficiency relation between i and j can be explained by the statistical dependence induced through the common cause k. Therefore, the directional correlation between i and j is regarded as spurious.

From a different perspective, the Reichenbach-like test avoids Simpson-type paradoxes arising from the partition of the data according to the deregulation status of any common-cause gene.

S2.2 Mokken test of double monotony

Some links may be redundant due to causal transitivity if $i \rightarrow k$ is explained by the presence of $i \rightarrow j$ and $j \rightarrow k$. We prune these by appealing to the Mokken test of double monotony [24]. Indeed, whenever, $i \rightarrow j$, $j \rightarrow k$ and $i \rightarrow k$, we check whether the following conditions are satisfied:

c) $v_{ij} \leq v_{ik} \leq v_{jk}$, and

d) $v_{\sim i\sim j} \geq v_{\sim i\sim k} \geq v_{\sim j\sim k}$,

where $v_{\sim i\sim j} = 1 - v_i - v_{jjj} + v_{ij}$ is the frequency of co-occurring non-deregulations at genes i and j. If conditions c) and d) hold, the arc $i \rightarrow k$ is removed from the GDN. The connection between the Mokken test and causal transitivity follows from the equivalence of c) and d) with

e) $v_{ij\sim k} \leq v_{i\sim jk} \leq v_{\sim ijk}$, and

f) $v_{\sim i\sim jk} \geq v_{\sim ij\sim k} \geq v_{i\sim j\sim k}$.

Conditions e) and f) induce a hierarchical ordering among patterns with one and two deregulations, respectively, by comparing frequencies of co-occurring deregulations and non-deregulations among the three involved genes, such that patterns incompatible with the implied transitive causal structure become progressively less likely.

S2.3 Heuristic test

Additionally, a heuristic test, originally suggested by Mokken as well [24], is applied to define the orientation of links in cases where $v_i = v_j$ and $H_{ij} = H_{ji}$ (i.e., where both edges $i \rightarrow j$ and $j \rightarrow i$ exist), which have not been eliminated by the Reichenbach-like test.

The heuristic test, which we refer to as the robustness criterion, starts by estimating more stable deregulation profiles of the involved genes. We assume that repeated measurements of the expression pattern of the same gene across the same samples reduce stochastic fluctuations in the inferred deregulation status. Indeed, a more robust estimate of the underlying deregulation pattern can be obtained by intersecting the results of two independent expression measurements of the same gene across the samples. The ensuing deregulation pattern will be denoted by i' if the initial gene was i, and by j' if the initial gene was j. Conditional and unconditional deregulation frequencies are computed analogously for i' and j' as for genes obtained from a single-shot measurement. In this case, we resolve the ambiguity in the direction of causation between i and j by applying the following tests:

I) If $H_{i'j} \leq H_0$ or $H_{i'j} < H_{j'i}$, we remove the edge $i \rightarrow j$.

II) If $H_{j'i} \leq H_0$ or $H_{j'i} < H_{i'j}$, we remove the edge $j \rightarrow i$.

In cases where $H_{i'j} = H_{j'i} > H_0$,

III) If $H_{i'j'} \leq H_0$, we remove the edge $i \rightarrow j$.

IV) If $H_{j'i'} \leq H_0$, we remove the edge $j \rightarrow i$.

We could still have $H_{i'j'} = H_{j'i'} > H_0$, but this is an extremely rare case. In this situation, both $i \rightarrow j$ and $j \rightarrow i$ are kept. These criteria evaluate the robustness of the inferred causal sufficiency relations under noise-reduced conditions.

Since we do not have access to repeated measurements of a gene expression pattern across samples, we estimate the deregulation and co-occurring deregulation frequencies $v_{i'}$, $v_{i'j}$ and $v_{i'j'}$ entering the Loevinger coefficients in I)-IV) as follows:

$v_{i'} \approx v_{ik}\, v_i \,/\, v_k$

$v_{i'j} \approx v_{ij}\, v_{i'} \,/\, v_i$

$v_{i'j'} \approx v_{ij}\, v_{i'}\, v_{j'} \,/\, (\, v_i\, v_j)$

where k is chosen as the gene with deregulation pattern most similar to that of i among genes satisfying $k \rightarrow i$ or $i \rightarrow k$. Extended derivations and further discussion of Loevinger, Reichenbach, Mokken and robustness tests can be found in Refs. [21,22].

S3. Robustness analysis and permutation based negative control

We evaluated the stability of N-gene and T-gene sets, as well as T-GDN edges, under variations in cohort composition and size.

S3.1 Effect of cohort composition (fixed size: 52 normal + 52 tumor)

We generated 100 cohort replicates by randomly drawing 52 distinct tumor samples from the original bank of 499 tumors, keeping the full set of 52 normal samples. One replicate was used as reference to compute recall and precision.

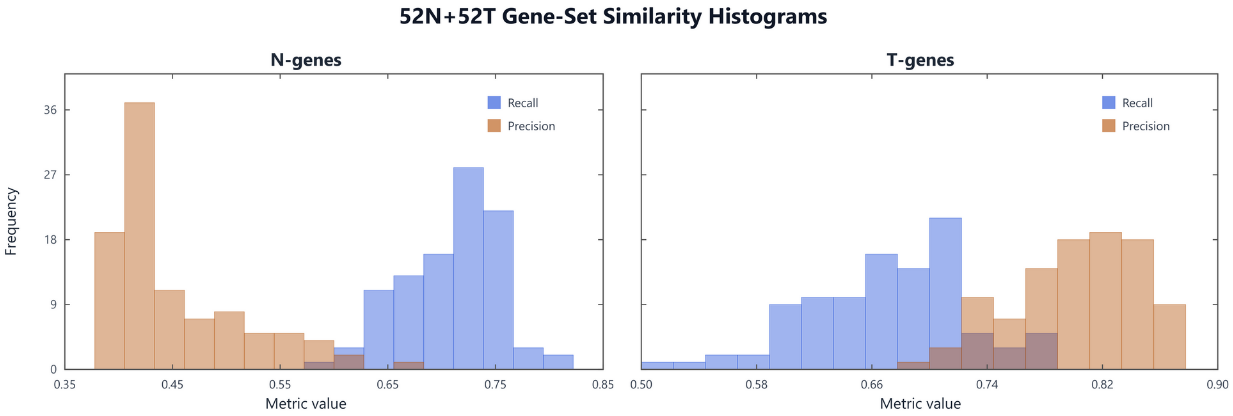

Figure S3.1. Stability of N- and T-gene discovery across original cohort resamplings. Histograms show the distribution of recall and precision values obtained from repeated 52 normal + 52 tumor real-cohort resampling experiments. Each replicate was compared against the selected 52N+52T reference cohort. The left panel shows N-gene set recovery and the right panel shows T-gene set recovery. Recall quantifies the fraction of reference genes recovered in each replicate, while precision quantifies the fraction of genes in each replicate that also belong to the reference set.

N-genes (left panel, Fig. S3.1):

Recall values are high (mode ~70%), but precision is low (mode <40%). This indicates that a sizable subset of N-genes (>70% of the reference set) is consistently detected across replicates. However, undersampling the tumor bank can create apparent exclusion intervals for non-N-genes, spuriously inflating replicate-specific N-gene sets and reducing precision.

T-genes (right panel, Fig. S3.1):

Both recall and precision are high (modes ~70% and ~80%, respectively). Unlike N-genes, T-genes are defined by tumor-exclusive expression intervals that may deplete upon undersampling. Nevertheless, broad tumor expression distributions preserve the relative proportion of tumor samples in those intervals, allowing consistent recovery. Moreover, undersampling cannot generate spurious T-genes because it cannot extend normal expression intervals. This explains the high precision.

S3.2 Effect of cohort size (increasing tumor samples)

We selected 10 independent replicates from the previous analysis (excluding the reference). Tumor samples were progressively added in increments of 52, up to 468 tumors, while keeping all normal samples.

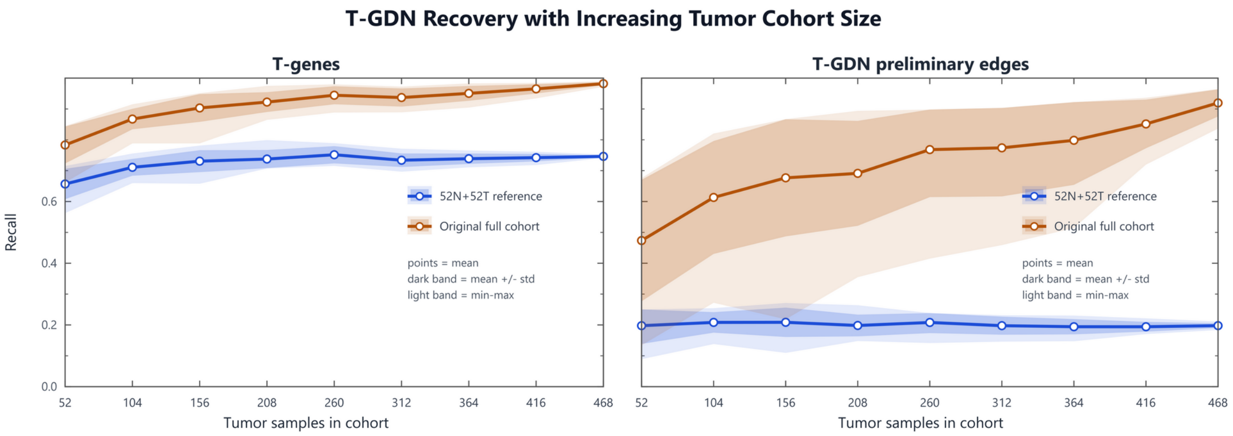

Figure S3.2. T-GDN structure recovery upon increasing tumor cohort size. Cumulative real-tumor augmentation was performed using 10 independent 52N+52T cohorts from the analysis leading to Fig. S1, excluding the 52N+52T reference cohort. For each sweep replicate, additional real tumors were sampled without replacement from the remaining tumor bank in increments of 52, up to 468 tumors. The left panel shows T-gene recall and the right panel shows preliminary T-GDN edge recall. Curves indicate the mean recall across sweep replicates; darker shaded bands indicate mean +/- standard deviation, and lighter shaded bands indicate the min-max range. Recovery is shown relative to both the held-out 52N+52T reference cohort and the original full-cohort reference.

T-gene recovery (left panel, Fig. S3.2):

Recall relative to the reference cohort (52 tumors) rapidly increases and saturates near 75%. Recall relative to the original full cohort (499 tumors) steadily increases, approaching 100% as the full tumor bank is recovered. With only half the tumor bank (~260 tumors), on average ~95% of the original T-genes are already recovered.

Edge recovery (right panel, Fig. S3.2):

Edges at the level of Stage 1 in Section 2.3 (no pruning) were computed. Approximately 20% of T-GDN edges from the reference cohort are consistently recovered regardless of sample size (stable core). Recall relative to the original cohort increases toward 100% as more tumors are added. Using half the tumor bank already recovers nearly 75% of the original edges.

Conclusion: The inferred GDNs are sufficiently stable to support the identification of candidate gene targets based on topological properties. Although nodes and edges may vary with cohort composition and size, a large portion of the network structure is consistently recovered by our gene and causal discovery algorithms.

S3.3 Permutation based negative control

We have performed the following permutation analysis to demonstrate that the resulting network structure in PRAD is not an artifact.

Procedure: synthetic binary deregulation matrices were generated by *independently* permuting sample labels for each gene. For a given gene, this procedure randomly reassigns the positions of the 1s and 0s across samples while preserving the total number of deregulated samples (1s), and thus the deregulation frequency, of that gene. Because the permutations are performed independently for each gene, the resulting matrices do not correspond to a simple global permutation of sample labels across genes. Applying the full T-GDN pipeline to 10 permuted datasets runs yields:

- Mean number of edges: 22 (vs. more than 100000 in real data)
- Mean edge density: 0.00005% (vs. 0.3%)
- No permuted dataset produced > 31 edges

This confirms that the observed network structure is not recoverable from random data with the same marginal frequencies.

Run	Number of edges	Edges in the original graph
1	30	0
2	24	0
3	22	0
4	21	0
5	17	0
6	18	0
7	31	0
8	19	0
9	20	0
10	17	0
Mean	21.9	0

S3.4 Robustness of N-gene block structure

We evaluated the stability of N-gene sets and their block structure under variations in cohort size. We generated 10 cohort replicates by randomly drawing a varying number of samples from the original bank of 52 normal samples, keeping the full set of 499 tumor samples. Normal samples were progressively added to each cohort replicate in increments of 10, up to 50 normal samples.

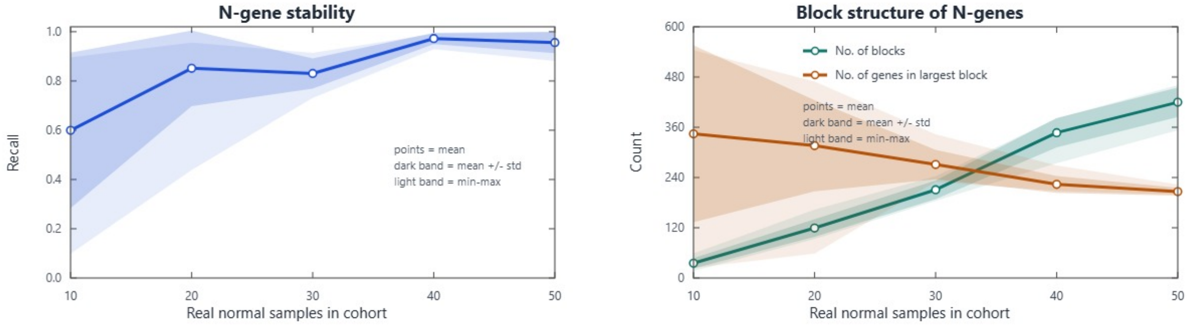

Figure S3.3. N-gene stability and block structure under real-normal subsampling. Original full-cohort N-genes were kept as the reference gene set and re-evaluated after constructing cohorts with increasing numbers of real normal samples, while keeping all original tumor samples fixed. Real normal samples were drawn without replacement for each replicate, using cohorts of 10 to 50 normal samples; the full 52-normal endpoint was not included because it corresponds to the original full-normal cohort. The left panel shows the recall of original N-genes that still satisfy the N-gene criteria after subsampling. The right panel shows the resulting block structure of the retained N-genes after cohort-specific discretization, where a block is defined as a set of genes sharing an identical binary pattern across the selected normal samples. Points indicate replicate means, dark shaded bands indicate mean +/- standard deviation intervals, and light shaded bands indicate the min-max range across replicates.

N-gene recovery (left panel, Fig. S3.3):

Recall relative to the original cohort (52 normal samples) decreases smoothly with decreasing normal-bank size, featuring two plateaus between 20–30 and 40–50 normal samples on average. The lowest recall value, reached for one-fifth of the normal bank, is ~60%.

N-gene block structure (right panel, Fig. S3.3):

The number of blocks decrease steeply with decreasing normal-bank size, from ~ 420 to ~ 35. However, the largest block increases with decreasing normal samples more slowly, from 212 for the original cohort size to ~344 genes for one-fifth of the normal bank.

Conclusion: N-gene recovery results confirm their stability with respect to changes in normal-bank size, even for very small numbers of normal samples. This robustness likely reflects the lower variance of the normal slice compared to the tumor slice [41]. We also observe some sample dependence in the block structure of N-genes, with the number of blocks decreasing substantially as the normal-bank size is reduced. Indeed, reducing the number of normal samples allows more genes to be merged into the same block. Nevertheless, the largest N-gene block remains relatively stable across changes in the normal slice. These results suggest that, although part of the block structure is sensitive to sample size, it cannot be entirely explained as a statistical artifact. See the main text for a positive hypothesis regarding the origin of the remaining robust block structure.
